# Randomized controlled trials of major oral traditional Chinese medicine preparations for postherpetic neuralgia: an evidence map

**DOI:** 10.3389/fphar.2026.1815376

**Published:** 2026-06-08

**Authors:** Kelin Li, Mingyue Liu, Baixiang He, Haojin Cheng, Jingyu Lv, Jishu Wang, Ping Song, Sheng Chen

**Affiliations:** 1 Dongzhimen Hospital, Beijing University of Chinese Medicine, Beijing, China; 2 Suzhou Hospital, Xiyuan Hospital of China Academy of Chinese Medical Sciences, Suzhou, China; 3 Graduate School, Beijing University of Chinese Medicine, Beijing, China; 4 Xiyuan Hospital, China Academy of Chinese Medical Sciences, Beijing, China; 5 Chengdu University of Traditional Chinese Medicine, Chengdu, China; 6 First Teaching Hospital of Tianjin University of Traditional Chinese Medicine, Tianjin, China; 7 National Clinical Research Center for Chinese Medicine, Tianjin, China; 8 Henan University of Chinese Medicine, Zhengzhou, Henan, China

**Keywords:** evidence mapping, methodological quality, postherpetic neuralgia, randomized controlled trial, traditional Chinese medicine

## Abstract

**Objective:**

To identify and synthesize randomized controlled trials (RCTs) of oral traditional Chinese medicine (TCM) for postherpetic neuralgia (PHN), map the volume, methodological quality, and evidence distribution, identify evidence gaps, and inform future research.

**Methods:**

We systematically searched PubMed, Embase, the Cochrane Library, China National Knowledge Infrastructure (CNKI), Wanfang Data, VIP Database, the Chinese Clinical Trial Registry (ChiCTR), and China Biomedical Literature Database (CBM). RCTs published from database inception to 1 August 2025 were included. The evidence was summarized using evidence maps and narrative synthesis. Risk of bias in the included RCTs was assessed using the Cochrane Risk of Bias tool (RoB 1.0).

**Results:**

A total of 357 RCTs were included, most were small studies with sample sizes ranging from 50 to 100 participants. Most studies reported diagnostic criteria and inclusion/exclusion criteria, however, limited attention was paid to TCM syndrome differentiation and its standardization. The main outcomes were pain degree, clinical effective rate, adverse reactions, sleep quality, negative emotions, and quality of life, however, outcomes such as recurrence and TCM syndrome scores were infrequently reported. Overall methodological quality was low, as assessed using the RoB 1.0.

**Conclusion:**

Although many included studies reported favourable findings, the formulas and preparations differed substantially in composition, and high-quality evidence remains limited. Future trials should better incorporate key features of TCM (e.g., syndrome differentiation), use standardized and clinically meaningful outcome measures, and strengthen trial design and reporting to reduce risk of bias and improve the credibility of evidence.

**Systematic Review Registration:**

URL: https://www.crd.york.ac.uk/PROSPERO/view/CRD420251110786, identifier CRD420251110786.

## 1 Introduction

Postherpetic neuralgia (PHN) is a chronic pain condition that develops after herpes zoster (HZ) and is the most common complication of HZ (Gross et al., 2020; Lim et al., 2024). Herpes zoster results from reactivation of the varicella-zoster virus (VZV), which remains dormant in sensory ganglia after primary varicella infection, PHN may subsequently develop as persistent neuropathic pain (Katsarakes et al., 2022). The pathogenesis of PHN remains incompletely understood (Liu et al., 2024; Yan et al., 2025). Maladaptive neuroplasticity is thought to contribute to PHN and may involve peripheral and central sensitization, inflammatory responses, and deafferentation (Johnson et al., 2007).

Although PHN is rarely life-threatening, severe pain can substantially impair quality of life. Approximately 40% of patients reported that neuropathic pain moderately or severely interfered with general activities, and nearly half reported moderate-to-severe anxiety or depression (Oster et al., 2005). A US study covering 1994–2018 reported an overall PHN incidence of 57.5 cases per 100,000 person-years (95% CI 56.0–59.0), the proportion of individuals with HZ who developed PHN was higher in 2007–2018 than in 1994–2006 (Thompson et al., 2021). In the United States, the annual direct medical costs and productivity losses attributable to HZ and its complications, including PHN, are estimated at approximately $2.4 billion (Harvey et al., 2020).

Current management of PHN typically involves a multimodal approach. A Chinese expert consensus recommends standardized management of PHN. Key principles include early initiation, adequate dosing, a sufficient treatment duration, and combination therapy when appropriate. The recommended first-line drugs include calcium channel regulators (pregabalin and gabapentin), tricyclic antidepressants (amitriptyline), and 5% lidocaine patches. Second-line options include opioids and tramadol (Yu et al., 2016). Recent advances, including herpes zoster vaccination, have shown promise in reducing the incidence of both HZ and PHN (Mbinta et al., 2022; Tseng et al., 2026).

However, an estimate suggests that up to 50% of patients with PHN may not respond adequately to treatment (Sacks, 2013). Both systemic and topical therapies are commonly associated with adverse effects, including dizziness, drowsiness, sedation, cardiotoxicity, and local burning or erythema. Opioid-based regimens also carry risks of misuse and dependence (Ngo et al., 2020). Patients with PHN, often older adults, commonly use five or more concomitant medications (Hadley et al., 2016).

Oral Chinese herbal medicine is a core component of traditional Chinese medicine (TCM) and has been used for centuries in China. In clinical practice in China, Chinese herbal medicine is often used alone or in combination with conventional therapies for PHN. A meta-analysis including 1,228 patients with PHN reported that oral Chinese herbal medicine plus pregabalin was associated with higher overall response and cure rates, lower pain scores, and improved sleep quality compared with pregabalin alone (Sang et al., 2024). In recent years, an increasing number of clinical trials have evaluated TCM interventions for PHN. Randomized controlled trials (RCTs) are the gold standard for evaluating interventions (Bothwell et al., 2016) and provide a key evidence base for guideline development and for addressing knowledge gaps. Therefore, rigorous appraisal of trial design, conduct, and reporting is essential to strengthen the evidence base in this field.

Methodologically, an evidence-mapping approach was used to characterize the clinical trial evidence on oral Chinese herbal medicine for PHN, including both quantitative and qualitative aspects. Unlike systematic reviews that address a specific question, evidence maps provide an overview of the volume, design, and key characteristics of studies within a topic area (Campbell et al., 2023; Khalil et al., 2025). Evidence mapping is typically used to summarize the evidence landscape, appraise methodological quality, and identify knowledge gaps to inform future research (Bragge et al., 2011; Probst et al., 2019).

Clinically, treatment selection for PHN should consider multiple factors, including efficacy, adverse effects, misuse potential, and cost. When monotherapy fails to achieve satisfactory control, adjunctive TCM may be considered as part of an individualized strategy to optimize symptom management. This evidence map visually summarizes RCTs of oral Chinese herbal medicine for PHN and assesses methodological quality, providing an accessible overview of the evidence landscape while recognizing the compositional diversity of the included interventions.

## 2 Methods

The protocol was registered in the International Prospective Register for Systematic Reviews (CRD420251110786), with no separate full protocol or amendments to the registration record. The evidence map was conducted and reported in accordance with PRISMA 2020 (Page et al., 2021a; Page et al., 2021b), and the search reporting followed PRISMA-S (Rethlefsen et al., 2021).

### 2.1 Data sources and searches

A comprehensive search was conducted in CNKI (China National Knowledge Infrastructure), Wanfang Data, VIP Database, SinoMed, the Chinese Clinical Trial Registry (ChiCTR), PubMed, Embase, and the Cochrane Library, from database inception to 1 August 2025. Chinese search terms included “带状疱疹后遗神经痛/带状疱疹后神经痛”, “缠腰火丹”, “蛇串疮”, “中医”,“中医药”,“中药”,“中西医结合”, “中药汤剂”, and “中成药”, among others. English search terms: Postherpetic Neuralgia, Traditional Chinese Medicine, Chinese Medicine, Chinese Drug, Traditional Drug, Traditional Herb, Chinese Patent Medicine, etc. The search strategy adopts a combination of free words and MeSH subject headings to improve accuracy. The complete search strategy is provided in the Supplementary Material.

### 2.2 Eligibility criteria

#### 2.2.1 Inclusion criteria

Population: Patients meeting diagnostic criteria for postherpetic neuralgia (PHN), with no restrictions on age, ethnicity, or sex.

Interventions: The intervention group received oral Chinese herbal medicine, including decoctions, granules, commercial Chinese polyherbal preparation, and herbal extracts, with no restrictions on formulation or dosage form. Chinese herbal medicine could be used alone or as an adjunct to other active interventions.

Comparison: Comparators included placebo, no treatment/usual care, or active interventions (e.g., oral medications, physical therapy, topical agents, injection therapy, or nerve block).

Study type: Randomized controlled trials (RCTs).

#### 2.2.2 Exclusion criteria

Studies with duplicate publications, insufficient data for extraction, or unavailable full texts were excluded. Studies were excluded if Chinese herbal medicine was combined with other interventions and the co-interventions were not identical between the intervention and control groups.

### 2.3 Study selection and data extraction

Retrieved records were imported into NoteExpress (v4.0.0) for deduplication. Two researchers independently screened titles/abstracts and then full texts according to the predefined eligibility criteria. Data were extracted using a piloted Microsoft Excel 2016 form and included: (1) general information (first author, publication year, country/region, journal, funding, etc.); (2) study characteristics (design, random sequence generation, allocation concealment, blinding, sample size); (3) population characteristics (age, sex, eligibility criteria, disease duration, disease severity, TCM syndromes); (4) intervention details (type, regimen components, frequency, and duration); and (5) outcomes (clinical efficacy rate, pain measures, serological markers, adverse reactions, quality of life, negative emotions, recurrence/relapse, etc.). Data extraction was performed independently by two reviewers (from a team of four), and discrepancies were resolved by discussion, unresolved disagreements were adjudicated by the lead investigator (Page et al., 2021a). Before formal screening and data extraction, 10% of eligible records were randomly selected for pilot testing. Discrepancies identified during pilot testing were discussed in a consensus meeting to refine the extraction form and minimize errors. When necessary, corresponding authors were contacted for missing or unclear information.

### 2.4 Botanical drug identification

According to the ConPhYMP guidelines (Heinrich et al., 2022), the characteristics of classic formulas that appeared more than 5 times in this review were described. Species names were checked against Plants of the World Online (POWO) following the approach of Rivera (Rivera et al., 2014), and drug names were standardised according to the Chinese Pharmacopoeia (Chinese Pharmacopoeia Commission, 2020). For the purpose of standardisation, the recorded compositions of classic formulas in this review did not include modified adjunctive botanical drugs. In routine TCM practice, such modifications are commonly used to address accompanying symptoms, while the core compatibility and principal therapeutic orientation of the original formula are generally preserved.

### 2.5 Methodological quality assessment

Risk of bias in included studies was assessed using the Cochrane Risk of Bias tool (RoB 1.0) (Higgins et al., 2011). The assessment covered seven domains: random sequence generation, allocation concealment, blinding of participants and personnel, blinding of outcome assessment, incomplete outcome data (attrition bias), selective reporting, and other sources of bias. Each domain was judged as “low risk,” “high risk,” or “unclear risk.” Disagreements were resolved by discussion; if consensus was not reached, adjudication was provided by the lead investigator.

### 2.6 Data synthesis and analysis

Descriptive statistics were presented in the form of text, figures and tables. Tables were used to present the botanical classification and other identification information of botanical drugs. Figures were generated using Origin 2024. The trend was represented by a line chart. Bar charts were used to display the distribution of risk-of-bias judgments across the seven RoB 1.0 domains. Bubble charts were used to visualize the distribution and methodological quality of evidence across different interventions and outcomes (Khalil et al., 2025).

## 3 Results

### 3.1 Study selection

**FIGURE 1:**
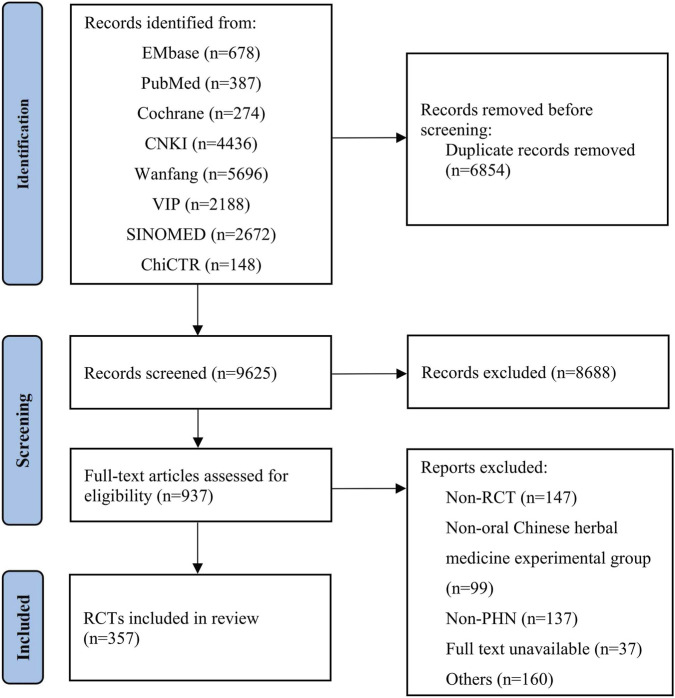
PRISMA flow diagram of literature selection process.

A total of 16,479 records were identified through database searching. After deduplication using automated and manual methods, 9,625 records remained. Titles and abstracts were screened, and 937 records were retained for full-text assessment. Full texts were assessed for eligibility, 580 records were excluded. Finally, 357 studies were included in the evidence map. The study selection process is shown in Figure 1.

### 3.2 Study characteristics

#### 3.2.1 Annual trends in publications

**FIGURE 2:**
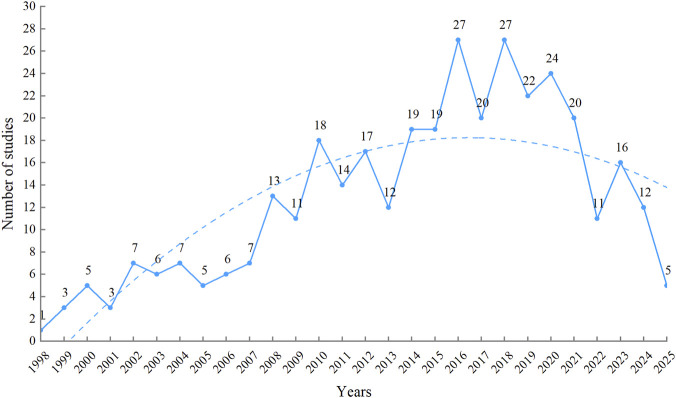
Annual trends in publications on RCTs on oral Chinese herbal medicine for PHN.

The included studies were published between 1998 and 2025, showing an overall fluctuating upward trend. Publication peaks occurred in 2016 and 2018 (28 studies each, 7.8%), followed by a decline after 2018. The annual publication trend is shown in Figure 2. The number of publications decreased markedly from 2020 to 2022, which may be partly attributable to disruptions in clinical trial conduct during the COVID-19 pandemic. All 357 included studies were published in Chinese. Thirty-five trials reported funding support (9.8%).

#### 3.2.2 Sample size

Across 357 studies, a total of 30,966 participants were enrolled. Sample sizes ranged from 37 to 394 participants per study. Most studies enrolled 50–99 participants (n = 250, 70.0%). The remaining studies enrolled 1–49 participants (n = 21, 5.9%), 100–149 participants (n = 65, 18.2%), 150–199 participants (n = 12, 3.4%), or ≥200 participants (n = 9, 2.5%). Only 4 studies reported a sample size calculation.

### 3.3 Participant characteristics of included RCTs

#### 3.3.1 Age

**FIGURE 3:**
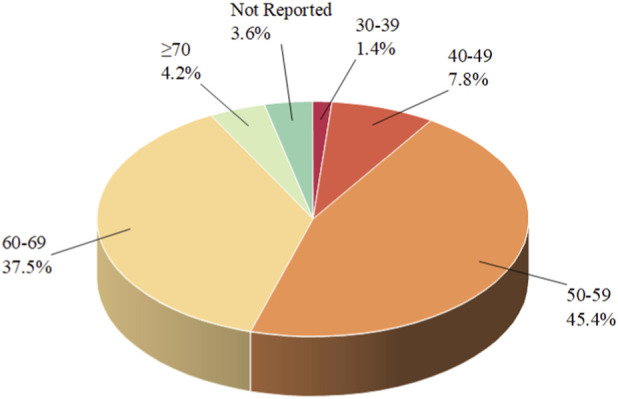
Proportion of patient age group.

Among all included RCTs (n = 357), 344 reported participant age (96.4%), whereas 13 did not report age. Mean age was extracted, when necessary, means and standard deviations were estimated from reported summary statistics (Wan et al., 2014). Stratified analyses showed that the reported mean age was 30–39 years in 5 trials (1.4%), 40–49 in 28 (7.8%), 50–59 in 162 (45.4%), 60–69 in 134 (37.5%), and ≥70 in 15 (4.2%). The age distribution of the patients is shown in Figure 3.

#### 3.3.2 Average VAS baseline

**FIGURE 4:**
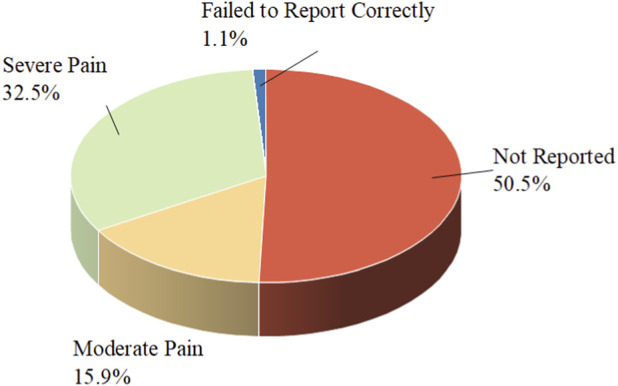
Proportion of patient’s pain levels.

Pain severity was categorized using the visual analog scale (VAS): 1–3, mild; 4–6, moderate; and 7–10, severe. Of the 357 included RCTs, 177 reported baseline mean VAS scores (49.6%). Among studies reporting baseline mean VAS, the mean score was 4 in 4 trials, 5 in 7 trials, 6 in 46 trials, 7 in 68 trials, 8 in 47 trials, and 9 in 2 trials. Accordingly, 57 trials mainly involved moderate pain (VAS 4–6) and 117 trials mainly involved severe pain (VAS 7–10). In addition, 4 articles failed to report the VAS score correctly. The distribution of pain severity is shown in Figure 4.

#### 3.3.3 TCM syndrome

**FIGURE 5:**
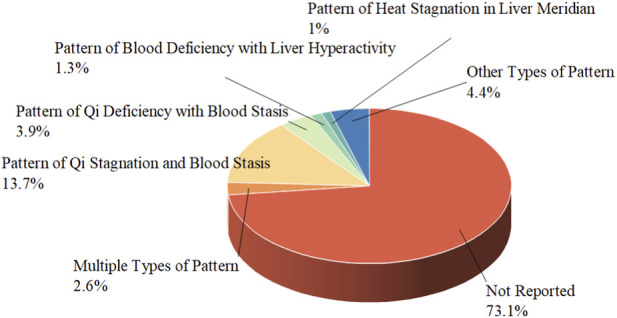
Proportion of different TCM sydrome.

Among the 357 RCTs, 104 reported TCM pattern differentiation (29.1%). Of these, 94 trials included participants with a single TCM pattern, whereas 10 trials included two or more patterns. The most frequently reported patterns were qi stagnation and blood stasis (n = 53, 14.8%), qi deficiency and blood stasis (n = 15, 4.2%), blood deficiency with liver hyperactivity (n = 5, 1.4%), and heat stagnation in the liver meridian (n = 4, 1.1%). Figure 5 shows the distribution of TCM pattern differentiation among the included RCTs.

### 3.4 Basic characteristics of the trial design in RCT

#### 3.4.1 Intervention characteristics of included RCTs

Oral TCM interventions in the intervention groups included decoctions prepared from Chinese botanical drugs (n = 312, 86.8%), TCM formula granules (n = 12, 3.4%), commercial Chinese polyherbal preparation (n = 26, 7.3%), and new traditional Chinese drug made from traditional Chinese medicine extracts (n = 7, 2.0%). In terms of composition, 10 trials used single-botanical drug products or extract-based preparations, including Bulleyaconitine A tablets (n = 2), compound glycyrrhizin (n = 2), Zhengqing Fengtongning sustained-release tablets (n = 2), Longxuejie capsules (n = 2), tanshinone capsules (n = 1), and centipede powder (n = 1). The remaining trials evaluated multi-botanical drug formulas. 11 classic formulas were used at least 5 times, including Xuefu Zhuyu Decoction (n = 37), Taohong Siwu Decoction (n = 31), Chaihu Shugan San (n = 15), Longdan Xiegan Decoction (n = 14), Fuyuan Huoxue Decoction (n = 12), Buyang Huanwu Decoction (n = 11), Shentong Zhuyu Decoction (n = 8), Shaoyao Gancao Decoction (n = 6), Xiaochaihu Decoction (n = 5), Xiaoyao San (n = 5), and Sini San (n = 5). A summary of these 11 classic formulas, together with detailed taxonomic and pharmacopoeial characterisation of their botanical drugs, is provided in Supplementary Table S1. When interpreting these evidences, some heterogeneity needs to be taken into account. For example, Xuefu Zhuyu Decoction is a substantially more complex multi-botanical-drug formula than Shaoyao Gancao Decoction, which contains only two constituent botanical drugs. Among these classic formulas, the most frequently used single botanical drugs are Chaihu (Bupleurum chinense DC. or Bupleurum scorzonerifolium Willd [Apiaceae; Bupleuri Radix]), Gancao (Glycyrrhiza uralensis Fisch. ex DC., or G. inflata Bat., or *G. glabra* L. [Fabaceae; Glycyrrhizae Radix et Rhizoma]) and Danggui (Angelica sinensis (Oliv.) Diels [Apiaceae; Angelicae Sinensis Radix]). Among the 26 trials using commercial Chinese polyherbal preparation, Xuefu Zhuyu-based products (capsules, oral liquid, tablets, or pills) were the most common (n = 11), accounting for 42.3%.

#### 3.4.2 Comparator characteristics of included RCTs

Control interventions included oral medications, injectable therapies, topical agents, physical therapy, nerve block, minimally invasive/regulatory techniques, acupuncture, placebo, and combinations of these approaches. Oral medication alone was the most common comparator (n = 225, 63.0%). The next most common comparator was oral medication plus injectable therapy (n = 46, 12.9%). Injectable therapy alone was used in 19 trials (5.3%), and oral medication plus physical therapy in 13 trials (3.6%). Only 1 trial used placebo as the comparator.

According to international and national clinical guidelines for neuropathic pain and herpes zoster-related pain, first-line pharmacotherapies for PHN include gabapentinoids (gabapentin and pregabalin), tricyclic antidepressants (e.g., amitriptyline and imipramine), and serotonin-norepinephrine reuptake inhibitors (e.g., duloxetine) (Attal et al., 2010; Finnerup et al., 2015; Expert Group for the Chinese Consensus on Diagnosis and Treatment of PHN, 2016). Among the 357 included RCTs, gabapentinoids were used in 103 trials, tricyclic antidepressants in 40 trials, and serotonin-norepinephrine reuptake inhibitors in two trials.

#### 3.4.3 Treatment duration

Among the 357 included RCTs, 349 reported treatment duration (97.8%). Treatment duration ranged from 5 days to 15 weeks. A duration of ≤4 weeks was reported in 235 trials (65.8%), 4–8 weeks in 95 trials (26.6%), and >8 weeks in 19 trials (5.3%).

### 3.5 Trial design characteristics of included RCTs

A total of 357 RCTs were included. Reported outcome measures were grouped into 10 categories: clinical effective rate (n = 333, 93.3%), pain outcomes (n = 187, 52.4%), adverse events (n = 169, 47.3%), sleep quality (n = 80, 22.4%), laboratory indicators (n = 48, 13.4%), negative emotions (n = 38, 10.6%), quality of life (n = 37, 10.4%), TCM syndrome scores (n = 24, 6.7%), symptom-related time outcomes (n = 14, 3.9%), and recurrence (n = 5, 1.4%). Clinical effective rate was treated as a study-defined composite outcome; its definition varied across studies but was generally based on symptom or pain improvement. Regarding pain assessment, 187 RCTs (52.4%) used validated pain rating scales. Among these, 171 trials (47.9%) used the Visual Analog Scale (VAS) alone and 9 trials (2.5%) used the Numerical Rating Scale (NRS) alone. The Verbal Rating Scale (VRS) was used in 2 trials, and the Present Pain Index (PPI) in 1 trial. Four trials used a combination of two or more pain scales.

Among the included RCTs, 58 trials (16.2%) reported follow-up, with the longest follow-up extending to 18 months after treatment completion. The most commonly reported follow-up time points were 1 month (n = 12) and 3 months (n = 16) after treatment. In addition, 24 trials (6.7%) reported dropout or loss to follow-up.

**FIGURE 6:**
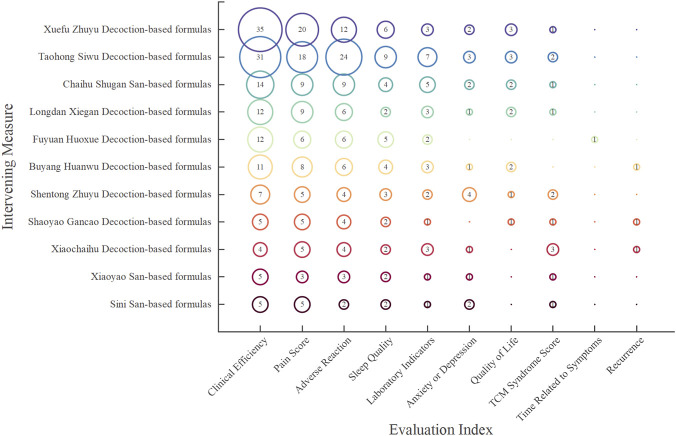
Distribution of evidence on evaluation metrics in clinical RCTs for the treatment of PHN with oral Chinese medicine. The size of each circle represents the number of publications that employed a particular evaluation metric for this intervention.

Figure 6 presents the distribution of outcome categories across TCM formulas used in ≥5 trials. The results indicate that clinical effective rate, pain outcomes, and adverse events were the most frequently reported outcomes, whereas recurrence, symptom-related time outcomes (e.g., time to pain relief), quality of life, and TCM syndrome scores were reported less often. Although Xuefu Zhuyu Decoction-based formulas were evaluated in the largest number of trials, these trials reported the fewest outcome categories per study on average. In contrast, trials of Xiaochaihu Decoction-based formulas and Sini San-based formulas reported more comprehensive outcome sets, with a greater number of outcome categories per study on average.

### 3.6 Quality assessment

**FIGURE 7:**
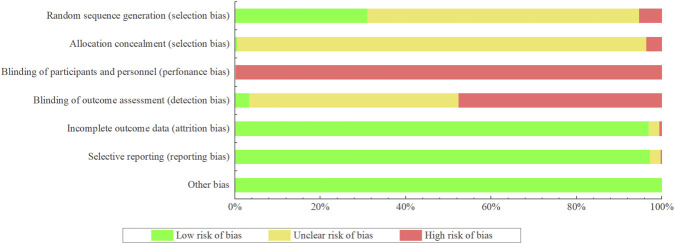
Summary of the risk of bias for RCTs.

Risk of bias in the 357 included RCTs was assessed using the RoB 1.0, as described in the Cochrane Handbook. The results showed that 133 trials described specific randomization methods. For random sequence generation, 111 trials (31.0%) were judged at low risk, 227 (63.6%) at unclear risk, and 19 (5.3%) at high risk. For allocation concealment, 2 trials (0.6%) were judged at low risk, 342 (95.8%) at unclear risk, and 13 (3.6%) at high risk. For blinding of participants and personnel, 1 trial (0.3%) was judged at low risk and 356 (99.7%) at high risk. For blinding of outcome assessment, 12 trials (3.4%) were judged at low risk, 175 (49.0%) at unclear risk, and 170 (47.6%) at high risk. For incomplete outcome data, 346 trials (97.0%) were judged at low risk, 9 (2.5%) at unclear risk, and 2 (0.6%) at high risk. For selective reporting, 347 trials (97.2%) were judged at low risk, 9 (2.5%) at unclear risk, and 1 (0.3%) at high risk. In this study, “other bias” was operationalized as conflicts of interest and was judged at low risk for all trials. The overall risk-of-bias judgments across all domains are summarized in Figure 7.

**FIGURE 8:**
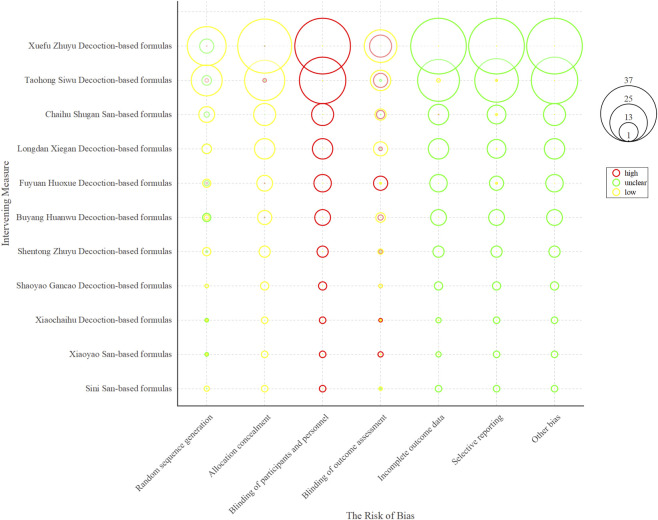
Evidence distribution of RCT methodology quality evaluation of oral Chinese medicine in treatment of PHN.

Figure 8 presents the risk-of-bias profile for traditional Chinese medicine compound prescriptions that were evaluated in ≥5 trials. Across these subgroups, the highest risk of bias was observed in blinding of participants and personnel, followed by blinding of outcome assessment and allocation concealment. The risk of bias in RCTs does not decrease due to the increased attention paid by researchers to specific traditional Chinese medicine compound prescriptions. In contrast, Sini San-based formulas showed the most favorable overall risk-of-bias profile.

## 4 Discussion

To our knowledge, this is the first evidence map to comprehensively characterize RCTs evaluating oral Chinese herbal medicine for PHN published up to 1 August 2025. A total of 357 RCTs were included. By synthesizing publication trends, sample sizes, participant characteristics, intervention/comparator features, outcome measures, and risk of bias, the evidence map provides an overview of the current evidence base. Integrating narrative synthesis with visual evidence maps provides an accessible overview of this research field and may help inform future trial design and reporting. These findings may help inform future trial design and reporting priorities in this field.

Although this review focused on clinical RCTs, several experimental studies provide preliminary mechanistic support for classic Chinese botanical drugs used in PHN and related neuropathic pain conditions. Taohong Siwu Decoction has been reported to alleviate PHN-related pain by suppressing neuroinflammation, neuronal apoptosis, and EphrinBs/EphBs signaling (Xu et al., 2021), and to improve peripheral nerve injury through PI3K/Akt/mTOR-related autophagy (Han et al., 2023). Xuefu Zhuyu Decoction has also shown potential analgesic and neuroprotective effects, possibly through inhibition of the NF-kappaB pathway (Liu et al., 2025) and regulation of the miR-191a-5p/BDNF-TrkB axis (Pei et al., 2024). In addition, some of the most frequently recurring botanical drugs across these classic formulas, particularly Chaihu (Bupleurum chinense DC. or Bupleurum scorzonerifolium Willd [Apiaceae; Bupleuri Radix]), Gancao (Glycyrrhiza uralensis Fisch. ex DC., or G. inflata Bat., or *G. glabra* L. [Fabaceae; Glycyrrhizae Radix et Rhizoma]), have also shown preliminary mechanistic relevance in related neuropathic pain models. Available studies suggest that their bioactive metabolites may exert anti-inflammatory, neuroprotective, and remyelination-promoting effects through pathways such as p38 MAPK/NF-κB (Dai et al., 2020), AQP1/RhoA/ROCK (Xiang et al., 2023), and Schwann cell-related signaling (Shi et al., 2022). These findings provide preliminary biological plausibility for some recurrent formulas used in PHN-related research, although direct mechanistic evidence remains limited. They should not be interpreted as supporting a single unified mechanism for all oral Chinese herbal medicine interventions.

### 4.1 Inconsistent diagnostic criteria for PHN

Diagnostic criteria for PHN remain inconsistent across guidelines and clinical trials. The 2016 Chinese expert consensus defines PHN as pain persisting for ≥1 month after resolution of the herpes zoster rash (Expert Group for the Chinese Consensus on Diagnosis and Treatment of PHN, 2016). In contrast, the 2020 German S2k guideline defines PHN as pain persisting for ≥3 months after rash resolution (Gross et al., 2020). Among the 357 included RCTs, only 213 (59.7%) reported the diagnostic criteria used for PHN. The most frequently cited diagnostic sources were the 2016 Chinese expert consensus (n = 39) (Expert Group for the Chinese Consensus on Diagnosis and Treatment of PHN, 2016), *the Diagnostic and Therapeutic Criteria for TCM Diseases and Syndromes* issued by the State Administration of Traditional Chinese Medicine (n = 35) (State Administration of Traditional Chinese Medicine, 1994), and the textbook *Clinical Dermatology* (Zhao Bian, ed.) (n = 32) (Zhao, 2017). Harmonized and explicitly reported diagnostic criteria are essential for improving trial rigor and cross-study comparability. Nonetheless, diagnostic standards are often incompletely reported in the current literature. Future trials should prespecify and clearly report the PHN diagnostic definition (including the time threshold after rash resolution) and reference internationally recognized guidelines to enhance consistency and reproducibility.

### 4.2 Core advantages of TCM not prominently featured

Syndrome differentiation and treatment (Bian Zheng Lun Zhi) is the fundamental diagnostic and therapeutic principle of the TCM theoretical system. It shapes the understanding of the etiology and pathogenesis of diseases, providing personalized treatment based on individual conditions. This process is crucial for demonstrating the clinical advantages of TCM. However, our study found that among all RCTs on TCM for PHN, only 104 trials (29.1%) conducted standardized TCM syndrome differentiation, the remaining studies did not specify the TCM syndrome patterns of the patients. Among the 104 trials that performed TCM syndrome differentiation, 74 explicitly cited reference standards for their syndrome patterns.

PHN is not explicitly defined in ancient TCM texts and is generally categorized as “Bi-syndrome,” primarily characterized by pain. In TCM theory, the etiology and pathogenesis of pain are typically categorized into two types: those caused by blockage in the body, and those resulting from deficiencies in nourishment, each requiring distinct therapeutic approaches. Furthermore, various syndrome patterns can manifest due to changes in disease etiology or individual constitutional differences, including excess syndromes (e.g., stagnated fire, damp-heat, blood stasis), deficiency syndromes (e.g., vital qi deficiency, yin deficiency with effulgent fire, or deficiency of qi and yin), and complex syndromes combining both excess and deficiency (e.g., qi deficiency with blood stasis, or yin deficiency with blood stasis). Accurate syndrome differentiation and pattern-specific prescriptions are fundamental to the efficacy of Chinese medicine. Clearly reporting the basis for TCM syndrome differentiation improves the scientific value and reference quality of experimental results (Cheng et al., 2017).

Moreover, evaluating efficacy based on TCM syndrome differentiation is a unique method that more accurately reflects the therapeutic advantages of Chinese medicine. Among the outcome measures, only 24 trials (6.7%) used TCM syndrome scores and reported changes. In standardized TCM clinical research, incorporating TCM syndrome scores as a core efficacy outcome not only reflects key theoretical principles of TCM but also strengthens the interpretability and evidentiary value of trials of oral TCM interventions. The absence of syndrome-based outcomes can perpetuate key methodological limitations in TCM trials, including heterogeneous evaluation criteria, limited quantitative assessment, and insufficiently objective measurement approaches, thereby constraining the ability to demonstrate TCM-specific benefits in a reproducible manner.

In summary, our findings indicate substantial room for improvement in the standardisation of both syndrome differentiation and syndrome-based outcome evaluation in clinical research on PHN. Future trials should report patients’ TCM syndrome patterns as essential baseline data and incorporate TCM syndrome scores as primary or secondary efficacy outcomes (Luo et al., 2015). To make this more actionable, future researchers should consider adopting explicit and validated syndrome differentiation criteria from national guidelines, textbooks, or expert consensus documents, and pre-specify the corresponding syndrome scoring methods in study protocols. In addition, stratified randomisation according to major TCM syndrome patterns, or at minimum prespecified subgroup analyses based on syndrome differentiation, may help improve baseline comparability and better reflect the principles of Bian Zheng Lun Zhi in trial design.

### 4.3 Low methodological quality

Among the included trials, only 111 reported an appropriate randomization method, only two described allocation concealment, only one reported blinding of participants, and 12 reported blinding of outcome assessment. For incomplete outcome data, 344 trials were judged at low risk (e.g., dropout <5% or intention-to-treat analysis). Selective reporting was judged at low risk in 345 trials. Overall, the evidence base is characterized by substantial risk of bias-particularly due to inadequate or incompletely reported randomization, allocation concealment, and blinding of participants and outcome assessors. Only one trial reported participant blinding; in that study, the intervention was delivered as tablets (Bulbus Aconiti Kusnezoffii), for which a matching placebo is relatively feasible (Koonrungsesomboon et al., 2024). By contrast, producing indistinguishable placebos for traditional decoctions can be particularly challenging, which may limit the feasibility of participant blinding in practice. Although placebo design for Chinese herbal formula granules remains challenging because of their characteristic colour, taste, and smell, compared with decoctions, formula granules may be more amenable to the development of matching placebos and may therefore represent a practical methodological option for future blinded trials. Therefore, when participant blinding is infeasible, priority should be given to rigorous randomization, robust allocation concealment, and blinded outcome assessment to mitigate bias.

These issues reflect broader challenges in trials of oral Chinese herbal medicine, including incomplete reporting and limited standardization of intervention protocols and implementation procedures. Consequently, the current evidence may be insufficiently robust to support reliable clinical decision-making (Wang et al., 2024; Salazar et al., 2025). Notably, several better-designed RCTs published in recent years suggest that higher-quality trials in this field are feasible and can yield clinically informative evidence. A rigorous appraisal of the existing evidence is therefore essential to identify key design and reporting gaps and to guide the development of more methodologically robust future trials.

### 4.4 Study limitations and future directions

This study integrates a large body of clinical evidence on oral Chinese herbal medicine for PHN and innovatively employs an evidence map for visual presentation. However, this study still has limitations: First, the evidence was limited to RCTs. Future research could also incorporate other study types, such as observational studies, non-randomized controlled trials, and meta-analyses, to broaden the scope of evidence and provide a more comprehensive overview of this field. Second, this review focused specifically on oral Chinese medicine and therefore did not include other important TCM modalities for PHN. In clinical practice, however, acupuncture and moxibustion-related therapies are also widely applied as part of the broader TCM approach to pain management. For example, a systematic review and meta-analysis of moxibustion for PHN included 13 RCTs involving 798 patients and reported potential benefits in clinical efficacy rate and pain reduction (Wu et al., 2021). In addition, recent multicentre study protocols have been published for electroacupuncture in patients with PHN and for intradermal acupuncture in acute herpes zoster with follow-up for PHN-related outcomes (Hu et al., 2021; Hu et al., 2023), reflecting ongoing efforts to generate more rigorous evidence across the herpes zoster–PHN continuum. Third, future studies should adopt standardised descriptions of Chinese materia medica, including botanical classification, species nomenclature, and medicinal parts used, to improve the clarity, reproducibility, and international interpretability of related research. Relevant frameworks such as the ConPhyMP guidelines (Heinrich et al., 2022) may serve as useful references. Another limitation is that the included trials rarely reported potential herb–drug interactions. As oral Chinese herbal medicine was often administered alone or in combination with other active interventions, future studies should report interaction-related safety more explicitly.

## 5 Conclusion

This study reviewed 357 RCTs literature on the treatment of PHN with oral Chinese herbal medicine. Referring to the PICOS principle in evidence-based medicine, the characteristics of the literature search results, the characteristics of the RCT research subjects, the intervention characteristics of the RCTs, and the result characteristics of the RCTs were sorted out and visualized. The methodological quality evaluation results were presented through a combination of text and icons, presenting the overall evidence of oral TCM in the treatment of PHN.

## Data Availability

The original contributions presented in the study are included in the article/Supplementary Material, further inquiries can be directed to the corresponding authors.
