# Supplementary material for: Randomized controlled trials of major oral traditional Chinese medicine preparations for postherpetic neuralgia: an evidence map
Source: Front Pharmacol. 2026 Jun 8;17:1815376. doi: 10.3389/fphar.2026.1815376 (PMC13311778; doi:10.3389/fphar.2026.1815376)
Supplement: Supplementary file 1 [file Table1.docx]

Supplementary Table S1. Taxonomic identification and pharmacopeial characterization of botanical drugs used in the 11 classic formulas appearing ≥5 times.

| Botanical drug  (Chinese name) | Validated species name [family; pharmacopeial drug name] | Part used | Weight ratio | Notes |
| --- | --- | --- | --- | --- |
| Xuefu Zhuyu Decoction | | | | |
| Chaihu (柴胡) | Bupleurum chinense DC. or Bupleurum scorzonerifolium Willd. [Apiaceae; Bupleuri Radix] | Dried root | 2 |  |
| Danggui (当归) | Angelica sinensis (Oliv.) Diels [Apiaceae; Angelicae Sinensis Radix] | Dried root | 6 |  |
| Taoren (桃仁) | Prunus persica (L.) Batsch or Prunus davidiana (Carr.) Franch. [Rosaceae; Persicae Semen] | Dried mature seed | 8 |  |
| Honghua (红花) | Carthamus tinctorius L. [Asteraceae; Carthami Flos] | Dried flower | 6 |  |
| Shengdihuang (生地黄) | Rehmannia glutinosa (Gaertn.) DC. [Orobanchaceae; Rehmanniae Radix] | Raw/dried root tuber | 6 |  |
| Chuanxiong (川芎) | Ligusticum chuanxiong Hort. [Apiaceae; Chuanxiong Rhizoma] | Dried rhizome | 3 |  |
| Chishao (赤芍) | Paeonia lactiflora Pall. or Paeonia veitchii Lynch [Paeoniaceae; Paeoniae Radix Rubra] | Dried root | 4 |  |
| Niuxi (牛膝) | Achyranthes bidentata Blume [Amaranthaceae; Achyranthis Bidentatae Radix] | Dried root | 6 |  |
| Jiegeng (桔梗) | Platycodon grandiflorus (Jacq.) A.DC. [Campanulaceae; Platycodonis Radix] | Dried root | 3 |  |
| Zhiqiao (枳壳) | Citrus × aurantium L. [Rutaceae; Aurantii Fructus] | Dried immature fruit | 4 |  |
| Gancao (甘草) | Glycyrrhiza uralensis Fisch. ex DC., or G. inflata Bat., or G. glabra L. [Fabaceae; Glycyrrhizae Radix et Rhizoma] | Dried root and rhizome | 2 |  |
| Taohong Siwu Decoction | | | | |
| Danggui (当归) | Angelica sinensis (Oliv.) Diels [Apiaceae; Angelicae Sinensis Radix] | Dried root | 3 |  |
| Chuanxiong (川芎) | Ligusticum chuanxiong Hort. [Apiaceae; Chuanxiong Rhizoma] | Dried rhizome | 2 |  |
| Baishao (白芍) | Paeonia lactiflora Pall. [Paeoniaceae; Paeoniae Radix Alba] | Dried peeled root | 3 |  |
| Shudihuang (熟地黄) | Rehmannia glutinosa (Gaertn.) DC. [Orobanchaceae; Rehmanniae Radix Praeparata] | Prepared root tuber | 4 |  |
| Taoren (桃仁) | Prunus persica (L.) Batsch or P. davidiana (Carr.) Franch. [Rosaceae; Persicae Semen] | Dried mature seed | 3 |  |
| Honghua (红花) | Carthamus tinctorius L. [Asteraceae; Carthami Flos] | Dried flower | 2 |  |
| Chaihu Shugan San | | | | |
| Chaihu (柴胡) | Bupleurum chinense DC. or Bupleurum scorzonerifolium Willd. [Apiaceae; Bupleuri Radix] | Dried root | 4 |  |
| Chenpi (陈皮) | Citrus reticulata Blanco and cultivars [Rutaceae; Citri Reticulatae Pericarpium] | Dried ripe pericarp | 4 |  |
| Chuanxiong (川芎) | Ligusticum chuanxiong Hort. [Apiaceae; Chuanxiong Rhizoma] | Dried rhizome | 3 |  |
| Xiangfu (香附) | Cyperus rotundus L. [Cyperaceae; Cyperi Rhizoma] | Dried rhizome | 3 |  |
| Zhiqiao (枳壳) | Citrus × aurantium L. and cultivars [Rutaceae; Aurantii Fructus] | Dried fruit (near-mature/older immature stage) | 3 |  |
| Baishao (芍药/白芍) | Paeonia lactiflora Pall. [Paeoniaceae; Paeoniae Radix Alba] | Dried peeled root | 3 |  |
| Zhigancao (炙甘草) | Glycyrrhiza uralensis Fisch. ex DC., G. inflata Bat., or G. glabra L. [Fabaceae; Glycyrrhizae Radix et Rhizoma] | Honey-processed dried root and rhizome | 1 |  |
| Longdan Xiegan Decoction | | | | |
| Longdancao (龙胆草) | Gentiana scabra Bunge, G. manshurica Kitag., G. triflora Pall., or G. rigescens Franch. [Gentianaceae; Gentianae Radix et Rhizoma] | Dried root and rhizome | 2 |  |
| Huangqin (黄芩) | Scutellaria baicalensis Georgi [Lamiaceae; Scutellariae Radix] | Dried root | 3 |  |
| Zhizi (栀子) | Gardenia jasminoides J.Ellis [Rubiaceae; Gardeniae Fructus] | Dried ripe fruit | 3 |  |
| Zexie (泽泻) | Alisma orientale (Sam.) Juzep. [Alismataceae; Alismatis Rhizoma] | Dried rhizome | 4 |  |
| Mutong (木通) | Akebia quinata (Thunb.) Decne., A. trifoliata (Thunb.) Koidz., or A. trifoliata subsp. australis (Diels) T.Shimizu [Lardizabalaceae; Akebiae Caulis] | Dried stem | 2 | Historically confused with aristolochic-acid-containing Guan Mutong(关木通); modern use should correspond to Akebiae Caulis. |
| Cheqianzi (车前子) | Plantago asiatica L. or P. depressa Willd. [Plantaginaceae; Plantaginis Semen] | Dried seed | 3 |  |
| Danggui (当归) | Angelica sinensis (Oliv.) Diels [Apiaceae; Angelicae Sinensis Radix] | Dried root | 1 |  |
| Shengdihuang (生地黄) | Rehmannia glutinosa (Gaertn.) DC. [Orobanchaceae; Rehmanniae Radix] | Raw/dried root tuber | 2 |  |
| Chaihu (柴胡) | Bupleurum chinense DC. or B. scorzonerifolium Willd. [Apiaceae; Bupleuri Radix] | Dried root | 2 |  |
| Gancao (甘草) | Glycyrrhiza uralensis Fisch. ex DC., G. inflata Bat., or G. glabra L. [Fabaceae; Glycyrrhizae Radix et Rhizoma] | Dried root and rhizome | 2 |  |
| Fuyuan Huoxue Decoction | | | | |
| Chaihu (柴胡) | Bupleurum chinense DC. or B. scorzonerifolium Willd. [Apiaceae; Bupleuri Radix] | Dried root | 5 |  |
| Gualougen / Tianhuafen (瓜蒌根/天花粉) | Trichosanthes kirilowii Maxim. or T. rosthornii Harms [Cucurbitaceae; Trichosanthis Radix] | Dried root | 3 |  |
| Danggui (当归) | Angelica sinensis (Oliv.) Diels [Apiaceae; Angelicae Sinensis Radix] | Dried root | 3 |  |
| Honghua (红花) | Carthamus tinctorius L. [Asteraceae; Carthami Flos] | Dried flower | 2 |  |
| Gancao (甘草) | Glycyrrhiza uralensis Fisch. ex DC., G. inflata Bat., or G. glabra L. [Fabaceae; Glycyrrhizae Radix et Rhizoma] | Dried root and rhizome | 2 |  |
| Chuanshanjia (穿山甲) | Manis pentadactyla Linnaeus [Manidae; historical Squama Manitis] | Scales (animal-derived; historical ingredient) | 2 | Not botanical.  Chuanshanjia was removed from the 2020 Chinese Pharmacopoeia; modern substitutions / regulatory handling vary. |
| Dahuang (大黄) | Rheum palmatum L., R. tanguticum Maxim. ex Balf., or R. officinale Baill. [Polygonaceae; Rhei Radix et Rhizoma] | Dried root and rhizome | 10 |  |
| Taoren (桃仁) | Prunus persica (L.) Batsch or P. davidiana (Carr.) Franch. [Rosaceae; Persicae Semen] | Dried mature seed | 5 |  |
| Buyang Huanwu Decoction | | | | |
| Huangqi (黄芪) | Astragalus membranaceus (Fisch.) Bunge or A. membranaceus var. mongholicus (Bunge) P.K.Hsiao [Fabaceae; Astragali Radix] | Dried root | 80 |  |
| Dangguiwei (当归尾) | Angelica sinensis (Oliv.) Diels [Apiaceae; Angelicae Sinensis Radix] | Dried root tail | 4 | Classical text specifies the root tail (gui wei), not the whole root. |
| Chishao (赤芍) | Paeonia lactiflora Pall. or P. veitchii Lynch [Paeoniaceae; Paeoniae Radix Rubra] | Dried root | 3 |  |
| Dilong (地龙) | Pheretima aspergillum (E. Perrier), Metaphire vulgaris (Chen), M. guillelmi (Michaelsen), or Amynthas pectiniferus (Michaelsen) [Megascolecidae; Pheretima] | Dried body (animal-derived) | 2 | Not botanical. |
| Chuanxiong (川芎) | Ligusticum chuanxiong Hort. [Apiaceae; Chuanxiong Rhizoma] | Dried rhizome | 2 |  |
| Taoren (桃仁) | Prunus persica (L.) Batsch or P. davidiana (Carr.) Franch. [Rosaceae; Persicae Semen] | Dried mature seed | 2 |  |
| Honghua (红花) | Carthamus tinctorius L. [Asteraceae; Carthami Flos] | Dried flower | 2 |  |
| Shentong Zhuyu Decoction | | | | |
| Taoren (桃仁) | Prunus persica (L.) Batsch or P. davidiana (Carr.) Franch. [Rosaceae; Persicae Semen] | Dried mature seed | 3 |  |
| Honghua (红花) | Carthamus tinctorius L. [Asteraceae; Carthami Flos] | Dried flower | 3 |  |
| Danggui (当归) | Angelica sinensis (Oliv.) Diels [Apiaceae; Angelicae Sinensis Radix] | Dried root | 3 |  |
| Niuxi (牛膝) | Achyranthes bidentata Blume [Amaranthaceae; Achyranthis Bidentatae Radix] | Dried root | 3 |  |
| Chuanxiong (川芎) | Ligusticum chuanxiong Hort. [Apiaceae; Chuanxiong Rhizoma] | Dried rhizome | 2 |  |
| Gancao (甘草) | Glycyrrhiza uralensis Fisch. ex DC., G. inflata Bat., or G. glabra L. [Fabaceae; Glycyrrhizae Radix et Rhizoma] | Dried root and rhizome | 2 |  |
| Moyao (没药) | Commiphora myrrha (T.Nees) Engl. and related Commiphora spp. [Burseraceae; Myrrha] | Gum resin | 2 |  |
| Wulingzhi (五灵脂) | Trogopterus xanthipes Milne-Edwards [Sciuridae; Trogopterorum Faeces] | Dried faeces (animal-derived) | 2 | Not botanical. |
| Dilong (地龙) | Pheretima aspergillum (E. Perrier), Metaphire vulgaris (Chen), M. guillelmi (Michaelsen), or Amynthas pectiniferus (Michaelsen) [Megascolecidae; Pheretima] | Dried body (animal-derived) | 2 | Not botanical. |
| Qianghuo (羌活) | Notopterygium incisum K.C.Ting ex H.T.Chang or N. franchetii H.de Boiss. [Apiaceae; Notopterygii Rhizoma et Radix] | Dried rhizome and root | 1 |  |
| Qinjiao (秦艽) | Gentiana macrophylla Pall., G. crassicaulis Duthie ex Burk., G. straminea Maxim., or G. dahurica Fisch. [Gentianaceae; Gentianae Macrophyllae Radix] | Dried root | 1 |  |
| Xiangfu (香附) | Cyperus rotundus L. [Cyperaceae; Cyperi Rhizoma] | Dried rhizome | 1 |  |
| Shaoyao Gancao Decoction | | | | |
| Baishao / Shaoyao (白芍/芍药) | Paeonia lactiflora Pall. [Paeoniaceae; Paeoniae Radix Alba] | Dried peeled root | 1 |  |
| Zhigancao (炙甘草) | Glycyrrhiza uralensis Fisch. ex DC., G. inflata Bat., or G. glabra L. [Fabaceae; Glycyrrhizae Radix et Rhizoma] | Honey-processed dried root and rhizome | 1 |  |
| Xiaochaihu Decoction | | | | |
| Chaihu (柴胡) | Bupleurum chinense DC. or B. scorzonerifolium Willd. [Apiaceae; Bupleuri Radix] | Dried root | 8 |  |
| Huangqin (黄芩) | Scutellaria baicalensis Georgi [Lamiaceae; Scutellariae Radix] | Dried root | 3 |  |
| Renshen (人参) | Panax ginseng C.A.Mey. [Araliaceae; Ginseng Radix et Rhizoma] | Dried root and rhizome | 3 |  |
| Zhigancao (炙甘草) | Glycyrrhiza uralensis Fisch. ex DC., G. inflata Bat., or G. glabra L. [Fabaceae; Glycyrrhizae Radix et Rhizoma] | Honey-processed dried root and rhizome | 3 |  |
| Banxia (半夏) | Pinellia ternata (Thunb.) Breit. [Araceae; Pinelliae Rhizoma] | Processed dried tuber | 3 |  |
| Shengjiang (生姜) | Zingiber officinale Roscoe [Zingiberaceae; Zingiberis Rhizoma Recens] | Fresh rhizome | 3 |  |
| Dazao (大枣) | Ziziphus jujuba Mill. [Rhamnaceae; Jujubae Fructus] | Dried fruit | 12 fruits |  |
| Xiaoyao San | | | | |
| Zhigancao (炙甘草) | Glycyrrhiza uralensis Fisch. ex DC., G. inflata Bat., or G. glabra L. [Fabaceae; Glycyrrhizae Radix et Rhizoma] | Honey-processed dried root and rhizome | 1 |  |
| Danggui (当归) | Angelica sinensis (Oliv.) Diels [Apiaceae; Angelicae Sinensis Radix] | Dried root | 2 |  |
| Fuling (茯苓) | Wolfiporia cocos (F.A.Wolf) Ryvarden & Gilb. [Polyporaceae; Poria] | Dried sclerotium | 2 |  |
| Baishao (白芍) | Paeonia lactiflora Pall. [Paeoniaceae; Paeoniae Radix Alba] | Dried peeled root | 2 |  |
| Baizhu (白术) | Atractylodes macrocephala Koidz. [Asteraceae; Atractylodis Macrocephalae Rhizoma] | Dried rhizome | 2 |  |
| Chaihu (柴胡) | Bupleurum chinense DC. or B. scorzonerifolium Willd. [Apiaceae; Bupleuri Radix] | Dried root | 2 |  |
| Shengjiang (生姜) | Zingiber officinale Roscoe [Zingiberaceae; Zingiberis Rhizoma Recens] | Fresh rhizome | 1 piece |  |
| Bohe (薄荷) | Mentha haplocalyx Briq. [Lamiaceae; Menthae Haplocalycis Herba] | Dried aerial parts | A small amount |  |
| Sini San | | | | |
| Zhigancao (炙甘草) | Glycyrrhiza uralensis Fisch. ex DC., G. inflata Bat., or G. glabra L. [Fabaceae; Glycyrrhizae Radix et Rhizoma] | Honey-processed dried root and rhizome | 1 |  |
| Zhishi (枳实) | Citrus × aurantium L. and cultivars [Rutaceae; Aurantii Fructus Immaturus] | Dried immature fruit | 1 |  |
| Chaihu (柴胡) | Bupleurum chinense DC. or B. scorzonerifolium Willd. [Apiaceae; Bupleuri Radix] | Dried root | 1 |  |
| Baishao / Shaoyao (白芍/芍药) | Paeonia lactiflora Pall. [Paeoniaceae; Paeoniae Radix Alba] | Dried peeled root | 1 |  |
