# Supplementary material for: Randomized controlled trials of major oral traditional Chinese medicine preparations for postherpetic neuralgia: an evidence map
Source: Front Pharmacol. 2026 Jun 8;17:1815376. doi: 10.3389/fphar.2026.1815376 (PMC13311778; doi:10.3389/fphar.2026.1815376)
Supplement: Supplementary file 2 [file Table2.docx]

Supplementary Table S2. The basic information of included randomized controlled trials.

| Title | Publication source | Study ID | First author | First author's affiliation |
| --- | --- | --- | --- | --- |
| The efficacy and safety of three methods for the treatment of postherpetic neuralgia | Journal of Guangxi Medical University | ZhaoYC 2017 | 赵玉丛 | Department of Dermatology, the Changping Hospital of Changping |
| 311nm紫外线联合血府逐瘀口服液治疗带状疱疹后神经痛的 临床疗效 | Chinese Journal of Practical Nervous Diseases | LiuY 2016 | 刘勇 | Department of respiratory medicine, Baoding second central hospital |
| TDP照射配合中草药治疗带状疱疹后神经痛疗效观察 | Hebei Medical Journal | WangMQ 2012 | 王美芹 | Halixun International Peace Hospital |
| 阿米替林联合中草药治疗带状疱疹后神经痛39例 | Clinical Medicine | WangLQ 2010 | 王来群 | 河南宏力医院皮肤科 |
| 辨证活血养阴法对45例带状疱疹后遗神经痛患者治疗效果观察 | Chinese Journal of Ethnomedicine and Ethnopharmacy | YanJ 2011 | 严静 | Guiyang Sixth Hospital |
| 补肾通络方治疗带状疱疹后遗神经痛４１例 | Chinese Medicine Modern Distance Education of China | LiangC 2013 | 梁成 | 河北北方学院附属第二医院 |
| 补阳还五汤合芍药甘草汤加减治疗带状疱疹后遗神经痛的疗效观察 | 东方药膳 | NiuXL 2021 | 牛晓丽 | 山西省长治市第二人民医院 中医老年病科 |
| Buyang Huanwu Decoction Treat Shingles Residual Neuralgia 70 Cases | Journal of Zhejiang Chinese Medical University | ZhouJF 2010 | 周建飞 | 浙江省文成县中医院 |
| 补阳还五汤加减治疗带状疱疹后遗神经痛的临床疗效 | 临床合理用药 | WuYQ 2021 | 吴幼清 | 福建省宁德市中医院皮肤科 |
| 补阳还五汤联合常规西药治疗老年带状疱疹后遗神经痛临床研究 | 新中医 | WangF 2020 | 王锋 | 郑州市第九人民医院麻醉科 |
| 补阳还五汤在带状疱疹后遗神经痛治疗中的疗效分析 | CJCM 中医临床研究 | YuHY 2020 | 于晖曜 | 常州市金坛区中医医院 |
| 补阳还五汤治疗带状疱疹后遗神经痛50例 | 陕西中医 | WangLX 2009 | 王丽霞 | 河北省秦皇岛市耀华医院 |
| 参芪消毒饮辅助治疗老年性带状疱疹的临床观察 | Guiding Journal of Traditional Chinese Medicine and Pharmacy | FanSH 2015 | 范淑红 | Affiliated Hospital of Inner ongolia Medical University |
| 草乌甲素片联合加巴喷丁治疗带状疱疹后神经痛的临床疗效 | 中华医学杂志 | JinYY 2021 | 金雨颖 | 上海交通大学医学院附属新华医院疼痛科 |
| Clinical Efficacy of Chaihu Guizhi Decoction Combined with Interferon in theTreatment of Postherpetic Neuralgia and lts Effects on Visual Analogue Scale Scoreand Sleep Quality | Evaluation and Analysis of Drug-Use in Hospitals of China | XuXY 2022 | 徐雪怡 | Dept.of Medical Rehabilitation, Hainan Provincial Anning Hospital |
| Observation on the Curative Effect of using Chaihu Shugan Powder Combined with ModifiedTaohong Siwu Decoction and Oxcarbazepine in Treating Postherpetic Neuralgia | Clinical Journal of Traditional Chinese Medicine | LiXY 2020 | 李秀英 | Anhui University of Chinese Medicine |
| Analysis on Treating 120 Cases of Postherpetic Neuralgia with ChaiHu ShuGan Powder and Modified TaoHong SiWu Tang | Western Journal of Traditional Chinese Medicine | ZhangY 2017 | 张永 | Liquan County TCM Hospital |
| Effects of Chaihu Shugan powder combined with gabapentin on pain efficacy,inflammatory index, pain-related substances and neuro-related factors in patientswith PHN | Chinese Journal of Hospital Pharmacy | WangNN 2019 | 王楠楠 | Second department of Neurology,Second affiliated Hospital of Qiqihar Medical College |
| Clinical Observation of Daitong Prescription for Post-herpetic Neuralgia | New Chinese Medicine | ChenHJ 2018 | 陈惠娟 | New Chinese Medicine |
| 带状疱疹后神经痛的中药治疗 | 白求恩军医学院学报 | HanB 2003 | 韩冰 | 石家庄市中医院 |
| 带状疱疹后遗神经痛采用消痛汤联合阿昔洛韦治疗的临床观察 | 中医中药 | JiangL 2014 | 姜蕾 | 山东中医药高等专科学校中医系 |
| 柴胡疏肝散联合普瑞巴林治疗带状疱疹后遗神经痛疗效观察 | 湖北中医药大学硕士论文 | SongHL 2018 | 宋海玲 | 湖北中医药大学 |
| 带状疱疹后遗神经痛治疗方案优选研究 | 山东中医药大学 | LiangJM 2004 | 梁俊梅 | 山东中医药大学 |
| 带状疱疹后遗神经痛中西医治疗 | 心理医生 | WangQ 2016 | 王群 | 成都市双流区中医医院 |
| 丹红柴芍汤配合西药治疗带状疱疹后遗神经痛34例 | 陕西中医 | ShiJ 2010 | 石军 | 宁夏自治区石嘴山市第一人民医院皮肤科 |
| 丹栀逍遥散加减联合伐昔洛韦治疗带状疱疹遗后神经痛30例 | Inner Mongolia Journal of Traditional Chinese Medicine | ChengGL 2014 | 程甘露 | 南阳医学高等专料学校 |
| 调气和血法治疗带状疱疹后遗神经痛50例临床观察 | 江苏中医药 | WangH 2008 | 王恒 | 上海交通大学附属仁济医院崇明分院 |
| 独活寄生汤加减治疗带状疱疹后遗神经痛30例临床观察 | 中医学报 | HongW 2010 | 洪文 | 广州市东升医院 |
| 分析桃红四物汤加减治疗带状疱疹后遗神经痛气滞血瘀证的临床疗效及体会 | 健康必读 | ZhuXJ 2019 | 朱霞俊 | 成都市金牛区沙河源社区卫生服务中心 |
| Analysis of Chinese herbal formula Qutong Tongluo Decoction for postherpetic neuralgia | Health for Everyone | KangXY 2020 | 亢新玉 | Lintao County People's Hospital |
| 扶正解毒通络法治疗带状疱疹后遗神经痛46例 | Journal of Anhui University of Chinese Medicine | ZhuBG 2001 | 朱波刚 | Second People's Hospital of Bengbu City |
| 复方甘草酸苷对带状疱疹后遗神经痛治疗效果的临床疗效探究 | 医学美学美容 | ZhengDM 2020 | 郑冬鸣 | 江苏省响水县人民医院皮肤科 |
| 复方甘草酸苷片治疗带状疱疹后遗神经痛的疗效 | 实用临床医学 | WuGG 2010 | 吴国根 | 南昌县人民医院皮肤科 |
| 复元活血汤加味治疗中重度带状疱疹后遗神经痛疗效及对疼痛相关神经肽与炎性因子的影响 | 现代中西医结合杂志 | LiJX 2022 | 利嘉欣 | 广东省第二中医院 |
| 复元活血汤联合超短波治疗带状疱疹后遗神经痛45例 | 陕西中医 | HouLJ 2013 | 侯立军 | 河北省邢台市中医医院 |
| 复元活血汤联合西药治疗带状疱疹后遗神经痛的疗效评价 | 中国农村卫生 | ChenF 2018 | 陈芳 | 北京市朝阳区中医医院皮肤科 |
| 复元活血汤联合西药治疗带状疱疹后遗神经痛的疗效评价 | 中外女性健康研究 | LiZF 2017 | 李志凡 | 辽宁省凌源市中心医院中医科 |
| 复元活血汤联合西药治疗带状疱疹后遗神经痛临床研究 | 新中医 | LiuDF 2019 | 刘丹凤 | 舟山市普陀区人民医院 |
| 复元活血汤治疗带状疱疹后遗神经痛的疗效观察 | 现代中西医结合杂志 | YangMF 2012 | 杨敏芳 | 广东省广州市花都区人民医院 |
| 复元活血汤治疗带状疱疹后遗神经痛的临床观察 | 北京中医药大学 | LinQS 2018 | 林琦珊 | 北京中医药大学 |
| 复元活血汤治疗带状疱疹后遗神经痛的效果探讨 | 临床医学 | GaoYH 2021 | 高彦洪 | 贵州省贵阳市修文县人民医院 |
| 瓜蒌红花甘草汤加减治疗带状疱疹后遗神经痛临床疗效观察 | 临床医药文献杂志 | ZhangZ 2018 | 张琢 | 内蒙古林业总医院中西医结合科 |
| 观察自拟四虫益气养血汤联合普瑞巴林治疗带状疱疹后遗神经痛的临床疗效及对日常生活质量、T淋巴细胞亚群水平的影响 | 养生保健指南 | ShanJH 2021 | 单建华 | 广州中研皮肤病专科门诊部 |
| Therapeutic Observations of Huayu Dingtong Tang in Treating PostherpeticNeruralgia due to Stagnancy of qi and Blood Stasis:A Report of 45 Cases | Henan Traditional Chinese Medicine | HuangYJ 2004 | 黄咏菁 | The Second Affiliated Hospital of Guangzhou University of Chinese Medicine |
| Study of Huayu Jiedu Decoction for postherpetic neuralgia (37 cases) | Beijing Journal of Traditional Chinese Medicine | JiaJM 2007 | JinmeiJia | Tianjin Yongjiu Hospital |
| 化瘀止痛汤治疗带状疱疹后遗神经痛的临床观察 | 河北中医 | LiuZG 2017 | 刘占国 | 河北省隆尧县医院中医科 |
| 黄芪桂枝五物汤合瓜红散加味治疗中老年带状疱疹后遗神经痛疗效观察 | 浙江中西医结合杂志 | XuJL 2017 | 许经纶 | 浙江省金华市第五医院皮肤科 |
| 黄芪桂枝五物汤加减治疗带状疱疹后遗神经痛的效果观察 | 宁夏医学杂志 | WangSH 2019 | 王寿海 | 宁夏银川市中医医院 |
| 活络效灵丹治疗带状疱疹后遗肋间神经痛30例 | 黑龙江中医药 | LiJS 2003 | 李俊松 | 黑龙江省大庆油田总医院 |
| 活络重镇汤治疗带状疱疹后遗神经痛 | 浙江中西医结合杂志 | SongLS 2006 | 宋力伟 | 浙江省丽水市中医院 |
| 活血化瘀法联合红光照射治疗带状疱疹后遗神经痛 | 中西医结合心血管病杂志 | FengY 2016 | 冯莹 | 陕西中医药大学 |
| 活血化瘀法治疗带状疱疹后遗神经痛48例 | 中医杂志 | ZhangZX 2002 | 张族祥 | 福建省三明市皮肤病医院宁化门诊部 |
| 活血化瘀法治疗老年带状疱疹后遗神经痛的临床分析 | 健康之友 | BaiJ 2018 | 白吉 | 山西省大同市第三人民医院皮肤科 |
| 活血化瘀法治疗老年带状疱疹后遗神经痛的临床效果 | 中国全科医学 | DingL 2018 | 丁丽 | 上海市第一人民医院宝山分院皮肤科 |
| 活血化瘀法治疗老年带状疱疹后遗神经痛的临床效果 | 中国医药指南 | WuCT 2017 | 吴长涛 | 辽阳市中医医院 |
| 活血化瘀法治疗老年带状疱疹后遗神经痛的临床效果研究 | 临床研究 | LiXL 2016 | 李学玲 | 甘肃省兰州石化总医院 |
| 活血化瘀法治疗老年带状疱疹后遗神经痛的临床研究 | 辽宁中医杂志 | WangLK 2016 | 王丽昆 | 华北理工大学附属医院皮肤科 |
| 活血化瘀通络法治疗带状疱疹后遗神经痛63例分析 | 浙江临床医学 | ZhangZH 2004 | 张兆和 | 浙江省青田县中医医院 |
| 活血解毒止痛方对带状疱疹后遗神经痛患者IL-6、IL-10水平的影响 | 人人健康 | CaoB 2021 | 曹彬 | 海安曹永泉皮肤科诊所 |
| 活血解毒止痛方治疗带状疱疹后遗神经痛疗效及对患者炎性因子的影响 | 陕西中医 | LuoHC 2018 | 罗恒超 | 河南省南阳市第二人民医院疼痛科 |
| 活血蠲痛汤联合西药治疗带状疱疹后遗神经痛临床观察 | 实用中医药杂志 | ShangJF 2016 | 商建飞 | 江苏省常州市中医医院麻醉科 |
| 活血祛瘀汤治疗带状疱疹后遗经痛的疗效观察 | 四川省卫生管理干部学院学报 | WangYZ 2008 | 王乙舟 | 简阳市中医院 |
| 活血散瘀汤对带状疱疹患者后遗神经痛的治疗效果分析 | 按摩与康复医学 | WangL 2020 | 王炼 | 宁德市疾病预防控制中心 |
| 解毒活血汤加减治疗老年带状疱疹后遗神经痛的疗效分析 | 临床医药文献杂志 | XuEH 2015 | 许恩宏 | 长春民族医院 |
| 解毒活血汤加减治疗带状疱疹后遗神经痛 63例 | 甘肃中医 | YangGD 2010 | 杨国栋 | 天水市中西医结合医院 |
| 净毒通络汤治疗带状疱疹后遗神经痛临床观察 | 四川中医 | LiJY 2016 | 李佳媛 | 成都中医药大学 |
| 了哥王片治疗带状疱疹后遗神经痛30例 | 浙江中医杂志 | GuZM 2002 | 顾仲明 | 浙江省江山市人民医院 |
| 六味地黄丸治疗带状疱疹后遗神经痛 | 光明中医 | ChenYW 2001 | 陈迎五 | 江苏省扬州市汶河医院 |
| 龙胆参麦止痉汤治疗带状疱疹后遗神经痛 32 例 | 实用中西医结合临床 | SunQJ 2017 | 孙清己 | 福建省宁德市中医院 |
| 龙胆泻肝软胶囊联合加巴喷丁治疗老年带状疱疹后遗神经痛临床观察 | 中国医学创新 | HuYE 2012 | 胡银娥 | 河南大学淮河医院 |
| 龙胆泻肝汤加减联合普瑞巴林治疗头面部带状疱疹后神经痛临床研究 | 新中医 | XuXY 2022 | 徐笑燕 | 淳安县中医院皮肤科 |
| 龙胆泻肝汤加减治疗带状疱疹后遗神经痛30例 | 中华皮肤科杂志 | WuSL 2000 | 吴胜利 | 上海市崇明县中心医院 |
| 龙胆泻肝汤加减治疗带状疱疹后遗神经痛的临床研究 | 上海医药 | YangJG 2015 | 杨建刚 | 上海市崇明县港西镇社区卫生服务中心 |
| 龙胆泻肝汤联合多柔吡星注射剂治疗带状疱疹后遗神经痛的疗效观察 | 医学临床研究 | CaoYC 2022 | 曹云超 | 陇县妇幼保健院中医科 |
| 龙胆泻肝汤联合脉冲射频治疗带状疱疹后遗神经痛的临床观察 | 陕西中医 | HuGR 2016 | 胡光瑞 | 吉林省中日联谊医院 |
| 龙胆泻肝汤联合普瑞巴林治疗带状疱疹后遗神经痛的效果分析 | 当代医药论丛 | DanYJ 2020 | 但杨军 | 大邑县人民医院 |
| 龙血竭胶囊治疗带状疱疹后遗神经痛25例疗效观察 | 新中医 | CaiHB 2008 | 蔡红兵 | 南方医科大学中医药学院 |
| 龙血竭胶囊治疗带状疱疹后遗神经痛 42 例疗效观察 | 医学信息 | JinGL 2016 | 金广连 | 江苏省邳州市人民医院 |
| 活血散瘀汤合阿昔洛韦治疗带状疱疹后遗神经痛疗效观察 | 现代中西医结合杂志 | GaoY 2018 | 高岩 | 北京市隆福医院 |
| 活血散瘀汤治疗带状疱疹后遗神经痛的临床观察 | 临床医药文献杂志 | XuFJ 2016 | 徐凤菊 | 江苏省响水县中医院 |
| 活血散瘀汤治疗带状疱疹后遗神经痛临床观察 | 实用中医药杂志 | LiZP 2018 | 李中平 | 江西中医药大学 |
| 活血散瘀止痛汤联合多虑平治疗带状疱疹后遗神经痛36例 | 中国中医药科技 | HeCW 2017 | 何成伟 | 浙江省宁波市奉化区人民医院 |
| 活血通络、疏肝理气、扶正固本汤治疗老年 PHN 的疗效观察 | 西南国防医药 | YuanJ 2015 | 袁晶 | 成都军区总医院高诊室 |
| 活血养血法治疗带状疱疹后遗神经痛疗效观察 | 世界中医药学会联合会皮肤病分会第二届年会 | ChenLH 2010 | 陈丽红 | 厦门市中医院皮肤科 |
| 加巴喷丁联合加味桃红四物汤治疗带状疱疹后遗神经痛56例疗效观察 | 饮食保健 | LiX 2018 | 厉鑫 | 东阳市中医院 |
| 加巴喷丁联合血府逐瘀汤对带状疱疹后遗神经痛患者的镇痛效果、 睡眠质量及预后的影响 | 贵州医药 | ZhangC 2018 | 张超 | 沈阳市第七人民医院皮肤科 |
| 加味丹栀逍遥散治疗带状疱疹后神经痛临床疗效分析 | 中华临床医学研究杂志 | TianG 2007 | 田刚 | 湖南省湘西自治州中医院皮肤科 |
| 加味金铃子汤合黛力新治疗带状疱疹后遗神经痛30例 | 浙江中医杂志 | LvZ 2008 | 吕振 | 浙江省宁波市中医院 |
| 加味四妙勇安汤治疗带状疱疹后遗神经痛 36 例临床观察 | 中国中西医结合皮肤性病学杂志 | ChenGQ 2010 | 陈国勤 | 广东省中医院珠海医院 |
| 加味桃红四物汤治疗带状疱疹后遗神经痛84例临床观察 | 中国中西医结合皮肤性病学杂志 | ZhangSB 2006 | 张少波 | 浙江省丽水市人民医院皮肤科 |
| 甲钴胺联合中药治疗顽固性带状疱疹后遗神经痛60例临床研究 | 当代医学 | WuWY 2016 | 吴文雅 | 陕西省澄合医疗中心王村矿医院 |
| 健脾散结汤辅助西医治疗中老年带状疱疹后遗神经痛疗效探讨 | 医药前沿 | ChenYH 2020 | 陈耀辉 | 绵阳市游仙区忠兴中心卫生院 |
| 解毒化瘀通络方治疗带状疱疹后遗神经痛毒损络瘀证疗效观察 | 中国中医急症 | KongYH 2011 | 孔宇虹 | 北京中医药大学东方医院 |
| 加味圣愈汤化裁治疗带状疱疹后遗神经痛90例 | 中国中医药科技 | DuCM 2012 | 杜长明 | 南京中医药大学 |
| 加味桃红四物汤治疗带状疱疹后遗神经痛的疗效观察 | 湖北中医杂志 | FangZQ 2011 | 方镇强 | 广州市番禺区慢性病防治站 |
| 普瑞巴林联合益气活血通络止痛方治疗带状疱疹后遗神经痛临床疗效观察 | 心理月刊 | ZengQQ 2020 | 曾清泉 | 甘肃省白银市景泰县中医医院 |
| 芪皮血府逐瘀汤治疗带状疱疹后遗神经痛疗效观察 | 陕西中医 | DingN 2015 | 丁宁 | 中铁二局集团中心医院中医科 |
| 秦马四虫汤加减治疗带状疱疹后遗神经痛95例疗效观察 | 河北中医 | ZhaoZJ 2013 | 赵湛君 | 河北省内丘县中医院皮肤科 |
| 祛痛方治疗带状疱疹后遗神经痛20例——附西药治疗20例对照观察 | 浙江中医杂志 | XuJ 2000 | 徐舰 | 浙江省杭州市第三人民医院 |
| 苗药止痛汤联合加巴喷丁治疗PHN的临床研究 | 贵州中医药大学 | CaoYY 2021 | 曹喻悦 | 贵州中医药大学 |
| 祛痛通络汤联合泛昔洛韦片治疗带状疱疹后遗神经痛临床观察 | 实用中医药杂志 | WuX 2020 | 吴潇 | 河南省南阳市第二人民医院疼痛科 |
| 祛痛通络汤联合西药治疗带状疱疹后遗神经痛60例 | 中医研究 | ZhangXD 2018 | 张晓丹 | 郑州市第一人民医院 |
| 祛痛通络汤治疗带状疱疹后遗神经痛60例 | 医学信息 | ZhangXK 2016 | 张小可 | 昌吉州中医医院 |
| 祛痛通络汤治疗带状疱疹后遗神经痛临床效果观察 | 世界最新医学信息文摘 | QingZY 2018 | 卿中亚 | 东莞市台心医院 |
| 袪痛方治疗带状疱疹后遗神经痛 | 山西中医 | ZhangJX 2012 | 张继霞 | 甘肃省临夏回族自治州人民医院 |
| 祛瘀镇痛汤加减治疗带状疱疹后遗神经痛临床疗效观察 | 湖北中医学院 | ChengJ 2009 | 程靖 | 湖北中医学院 |
| 全川散配合普瑞巴林治疗带状疱疹后遗神经痛疗效观察 | 皮肤病与性病 | ZhouSX 2019 | 周书祥 | 盐城市盐都区疾病预防控制中心 |
| 全蝎治疗带状疱疹后遗神经痛疗效观察 | 时珍国医国药 | WangQJ 2003 | 王启君 | 辽宁省海城市中医院 |
| 桃红四物汤加减治疗带状疱疹后遗神经痛42例 | 中西医结合与祖国医学 | LiHB 2012 | 李化冰 | 霍州煤电集团总医院 |
| 桃红四物汤加减治疗带状疱疹后遗神经痛58例 | 湖南中医杂志 | XueTP 2017 | 薛天萍 | 江苏省苏州市吴中人民医院 |
| 桃红四物汤加减治疗带状疱疹后遗神经痛的临床疗效观察 | 湖南中医药大学硕士学位论文 | LiXQ 2021 | 李晓沁 | 湖南中医药大学 |
| 桃红四物汤加减治疗带状疱疹后遗神经痛气滞血瘀证的临床疗效 | 内蒙古中医药 | SongLH 2021 | 宋丽华 | 鹤山市中医院 |
| 桃红四物汤加味联合红光治疗带状疱疹后遗神经痛疗效观察 | 辽宁中医药学报 | LuoCY 2014 | 罗春源 | 辽宁中医药大学 |
| 桃红四物汤加味治疗带状疱疹后遗神经痛26例 | 新中医 | WangWP 2009 | 王文平 | 宁复医科大学 |
| 桃红四物汤加味治疗带状疱疹后遗神经痛28例 | 现代中医药 | GuoHQ 2007 | 郭恒全 | 淳化县中医医院 |
| 桃红四物汤加味治疗带状疱疹后遗神经痛疗效观察 | 实用中西医结合临床 | QinZG 2016 | 秦治国 | 河南省鹤壁市淇县人民医院中西医结合科 |
| 桃红四物汤加味治疗老年带状疱疹后遗神经痛的疗效观察 | 光明中医 | WangPJ 2014 | 王朋军 | 河南濮阳市中医院皮肤科 |
| 桃红四物汤联合普瑞巴林治疗带状疱疹后遗神经痛疗效及机制研究 | 陕西中医 | QiaoSH 2019 | 樵书宏 | 陕西省商洛市中心医院皮肤科 |
| 桃红四物汤联合前列地尔治疗带状疱疹后遗神经痛的临床观察 | 湖南中医药大学学报 | XiaoJ 2017 | 肖杰 | 广西壮族自治区桂林市中医医院皮肤科 |
| 桃红四物汤联合双氯芬酸钠缓释胶囊治疗带状疱疹后遗神经痛效果观察 | 现代中西医结合杂志 | LiuLW 2017 | 刘立文 | 上海市普陀区人民医院 |
| 桃红四物汤联合双氯芬酸钠缓释胶囊治疗带状疱疹后遗神经痛的临床观察 | 中医药导报 | FuJ 2017 | 付静 | 简阳市中医医院 |
| 桃红四物汤治疗带状疱疹后遗神经痛的疗效及对Th1/Th2细胞因子的变化研究 | 中华中医药学刊 | YangXZ 2023 | 杨小珍 | 琼海市中医院 |
| 三虫通络汤联合加巴喷丁及神经阻滞治疗带状疱疹后遗神经痛的临床疗效观察 | WORLD CHINESE MEDICINE | ZhangY 2017 | 张英 | 西南医科大学附属中医院麻醉科 |
| 三合三虫汤治疗带状疱疹后遗神经痛 | 华夏医学 | ChenFX 2016 | 陈福祥 | 康美医院皮肤科 |
| 三种不同治疗方法对带状疱疹后遗神经痛的 临床疗效及安全分析 | 世界最新医学信息文摘 | YuZJ 2017 | 于志坚 | 武汉市江夏区第一人民医院疼痛科 |
| 散风清热组方治疗头面部带状疱疹后遗神经痛疗效观察 | 中国中西医结合皮肤性病学杂志 | ZhouZK 2018 | 周郅轲 | 岳阳市第二人民医院 |
| 芍药甘草汤加减治疗带状疱疹后遗神经痛的疗效观察 | 中国保健营养 | HanT 2022 | 韩韬 | 大连市中医医院皮肤科 |
| 芍药甘草汤加减治疗带状疱疹后遗神经痛对患者疼痛及血清NPY、SP水平的影响 | 海南医学 | LuXY 2021 | 陆星宇 | 西安宝石花长庆医院皮肤科 |
| 芍药甘草汤治疗带状疱疹后遗神经痛的临床疗效及其安全性 | 临床合理用药 | ZhouYP 2021 | 周银平 | 江西省上饶市玉山县人民医院 |
| 蛇丹愈后丸联合加巴喷丁治疗气虚血瘀型带状疱疹后遗神经痛的临床观察 | 中国实验方剂学杂志 | WangL 2018 | 王丽 | 河南省中医院 |
| 身痛逐瘀汤配合神经阻滞治疗带状疱疹后遗神经痛临床疗效及生活质量的影响 | 中国中医急症 | LiN 2016 | 李娜 | 西安医学院第二附属医院 |
| 身痛逐痛汤与多虑平联合在带状疙疹后遗神经痛中应用研究 | 辽宁中医药大学学报 | ZhangJ 2016 | 张坚 | 新疆维吾尔自治区人民医院 |
| 身痛逐瘀汤治疗老年人带状疱疹后遗神经痛67例 | 医学理论与实践 | HuangR 2004 | 黄榕 | 福建省汀州医院皮肤科 |
| 通络活血汤联合恩再适治疗带状疱疹后遗神经痛临床观察 | 中国现代医药杂 | WangSH 2008 | 王淑惠 | 河北省医科大学中医院皮肤科 |
| 通络祛痛汤治疗带状疱疹后遗神经痛 60 例疗效观察 | 新中医 | LiXS 2015 | 李喜顺 | 鹤煤集团公司总医院 |
| 通络益气方联合椎管内阻滞对带状疱疹后遗神经痛中医证候、 血清炎症因子、睡眠、负性情绪的影响 | 中华中医药学刊 | HouJQ 2022 | 侯俊青 | 浙江中医药大学滨江学院附属江南医院 |
| 通络益气方联合椎管内阻滞治疗PNH的临床疗效及其对血清炎症因子、 焦虑抑郁情绪的影响 | 世界中西医结合杂志 | LiangN 2020 | 梁男 | 成都医学院第一附属医院麻醉科 |
| 通络益气汤对带状疱疹后遗神经痛患者疼痛、睡眠及细胞免疫功能的影响 | 四川中医 | QiuSW 2020 | 邱旌伟 | 泸州市中医医院 |
| 身痛逐瘀汤联合多虑平治疗带状疱疹后遗神经痛临床疗效观察 | 湖北中医药大学硕士学位论文 | ChaJD 2013 | 查锦东 | 湖北中医药大学 |
| 神经痛口服液治疗带状疱疹后遗神经痛的疗效观察 | 航空航天医学杂志 | LiMJ 2011 | 李明杰 | 中航工业3201医院 |
| 神经阻滞联合复元活血汤治疗带状疱疹后遗神经痛 28例 | 中国中医急症 | ShenHF 2011 | 沈华芬 | 上海市仁济医疗集团余姚医院 |
| 神经阻滞联合中药治疗带状疱疹后遗神经痛临床疗效分析 | 医学综述 | XiaoYM 2007 | 肖宇民 | 湖南省中医院麻醉科 |
| 疏肝活血止痛方治疗带状疱疹后遗神经痛30例疗效观察 | 新中医 | FangYP 2009 | 方玉甫 | 河南省中医院皮肤科 |
| 疏肝解毒法治疗带状疱疹后遗神经痛90例临床观察 | 中国中医药科技 | SunJH 2010 | 孙剑虹 | 浙江省常山县人民医院 |
| 疏肝散火法内服治疗带状疱疹后神经痛的效果观察 | 智慧健康 | LiuMM 2019 | 刘苗苗 | 吉林市人民医院 |
| 疏肝散火法内服治疗带状疱疹后神经痛的效果观察 | 中国卫生产业 | JinYS 2012 | 金云顺 | 梅河口市中医院 |
| 疏肝散火法治疗带状疱疹后遗神经痛 30 例临床观察 | 医学信息 | LiL 2011 | 李雷 | 沅陵县中医院外科 |
| 疏肝益肾化瘀方治疗老年带状疱疹后遗症神经痛24例 | 陕西中医 | LiuYB 2009 | 刘毅斌 | 广西自治区柳州市人民医院中医科 |
| 疏肝逐瘀镇痛汤联合盐酸羟考酮缓释片治疗带状疱疹后遗神经痛的随机对照观察 | 中国中医药科技 | ZhaoLJ 2018 | 赵利军 | 山西医科大学第二医院 |
| 双花皂角汤联合西药治疗带状疱疹后遗神经痛的疗效及护理研究 | 心理医生杂志 | HanXL 2012 | 韩秀玲 | 山东省莱芜市人民医院 |
| 四虫益气养血汤联合普瑞巴林治疗带状疱疹后遗神经痛临床研究 | 陕西中医 | ZhuB 2019 | 祝波 | 湖北医药学院附属随州医院 |
| 四逆散合桂枝茯苓丸加味佐治带状疱疹后遗神经痛23例 | 国医论坛 | LinCH 2016 | 林春华 | 江门市人民医院 |
| 四逆散合桂枝茯苓丸治疗气滞血瘀型带状疱疹后遗神经痛45例 | 浙江中医杂志 | MaoCN 2020 | 毛春能 | 宁波市奉化区中医医院医共体 |
| 四物汤化裁治疗带状疱疹后遗神经痛疗效观察 | Clinical Journal of Traditional Chinese Medicine | ZhouXM 2011 | 周雪梅 | 安徽省萧县中医院皮肤科 |
| 汤药配合神经阻滞治疗带状疱疹后神经痛67例疗效观察 | 太传统医药 | LiFL 2011 | 李芳伶 | 赣州市人民医院 |
| 桃红四物汤合四逆散加味治疗带状疱疹后遗神经痛( 气滞血瘀证) 31 例 | 光明中医 | WangHY 2022 | 王浩悦 | 山西中医药大学 |
| 桃红四物汤加减方联合脉冲射频治疗带状疱疹后遗神经痛 | 中医学报 | HuYG 2020 | 胡艳阁 | 郑州市第六人民医院 |
| 通络止痛汤、加巴喷丁治疗带状疤疹后遗神经痛的临床疗效 | 中国麻风皮肤病杂志 | WangHS 2012 | 王海山 | 济钢总医院 |
| 通络止痛汤治疗带状疱疹后遗神经痛43例 | 中国中医急症 | SongQY 2012 | 宋秋云 | 浙江省奉化市中医院 |
| 头面部带状疱疹后遗神经痛的治疗体会 | 临床医药文献杂志 | GaoGJ 2019 | 高国娟 | 池州市第二人民医院 |
| 温阳活血汤加减联合普瑞巴林治疗带状疱疹后神经痛的临床观察 | 基层医学论坛 | DengTR 2020 | 邓田睿 | 大连市皮肤病医院 |
| 五苓散加味治疗带状疱疹后遗神经痛临床观察 | 内蒙古中医药 | ZhangLY 2014 | 张凌宇 | 辽宁省本溪市桓仁县中医院皮肤科 |
| 逍遥散加味治疗老年人带状疱疹后遗神经痛的临床效果 | 临床合理用药 | YangXD 2020 | 杨晓东 | 四川省巴中市巴州区三江镇卫生院 |
| 五味消毒饮辅治带状疱疹后遗神经痛临床观察 | 实用中医药杂志 | DengL 2021 | 邓璐 | 湖北省武汉市第一医院 |
| 消痛汤联合加巴喷丁胶囊治疗带状疱疹后遗神经痛临床观察 | 实用中医药杂志 | ZhouY 2020 | 周洋 | 河南省南阳市第二人民医院皮肤科 |
| 消痛汤配合阿昔洛韦治疗带状疱疹后神经痛疗效观察 | 中国实用医药 | LiuYC 2014 | 刘永春 | 河北省承德市丰宁满族自治县天桥镇卫生院中医科 |
| 小柴胡汤合瓜蒌红花散加减治疗带状疱疹后遗神经痛临床研究 | 新中医 | NiuZX 2021 | 钮正祥 | 桐乡市皮肤病防治院皮肤科 |
| 小柴胡汤加味治疗肝脾失和证带状疱疹后神经 痛临床研 究 | 云南中医药大学 | LiuYY 2021 | 刘于媛  | 云南中医药大学 |
| 小柴胡汤结合牵正散治疗带状疱疹后神经痛33例临床观察 | 湖南中医杂志 | XuHG 2018 | 徐浩刚 | 南京中医药大学附属江阴市中医院 |
| 小金丸联合加巴喷丁治疗带状疱疹后遗神经痛疗效观察 | 中国实用神经疾病杂志 | ZhangCH 2017 | 张朝晖 | 平煤神马医疗集团总医院皮肤科 |
| 小金丸治疗带状疱疹后遗神经痛44例 | 中国中医急症 | MaBH 2003 | 马保华 | 湖北襄樊市中医院 |
| 小金丸治疗带状疱疹后遗神经痛临床分析 | 数理医药学杂志 | LiHF 2018 | 李慧芳 | 河南省平顶山市平煤神马集团总医院皮肤科 |
| 星状神经节阻滞联合血府逐瘀胶囊治疗带状疱疹后神经痛的临床观察 | 中成药 | LinH 2006 | 林海 | 温州医学院附属第一医院麻醉科 |
| 行气活血通络治疗带状疱疹后遗神经痛126例观察 | 实用中医内科杂志 | YeWW 2006 | 叶文伟 | 浙江省青田县中医医院 |
| 血府逐瘀胶囊联合干扰素治疗带状疱疹后遗神经痛的临床研究 | 现代药物与临床 | WangY 2019 | 王宇 | 武汉市普仁医院 |
| 血府逐瘀胶囊联合LED红光照射治疗带状疱疹后遗神经痛疗效观察 | 中国实用神经疾病杂志 | DuanBX 2016 | 段宝学 | 武汉大学人民医院皮肤科 |
| 血府逐瘀胶囊联合红外线治疗老年人带状疱疹后遗神经痛效果的观察 | 国际老年医学杂志 | ZhangL 2012 | 张莲 | 长春中医药大学附属医院皮肤科 |
| 血府逐瘀胶囊联合照射窄谱中波紫外线治疗带状疱疹后遗神经痛得临床疗效 | 药物与临床 | ZhaoD 2015 | 赵丹 | 牡丹江医学院第二附属医院 |
| 血府逐瘀口服液联合高能窄谱红光照射治疗颌面部带状疱疹后遗神经痛疗效观察 | 实用中西医结合临床 | YiH 2015 | 易鸿 | 湖北省石首市骨科医院口腔科 |
| 血府逐瘀汤联合微波治疗带状疱疹后遗肋间神经痛的临床观察 | 湖北中医杂志 | LianAY 2010 | 练霭云 | 广东省清远市人民医院皮肤科 |
| 血府逐瘀汤联合红外线疗法治疗老年带状疱疹后遗神经痛的效果探究 | 当代医药论丛 | XuTY 2020 | 许天云 | 江油市许天云诊所 |
| 血府逐瘀汤联合LED红光治疗带状疱疹后遗神经痛临床疗效观察 | 内蒙古中医药 | WangJ 2015 | 王俊 | 四川省泸州市人民医院皮肤科 |
| 血府逐瘀汤加重镇安神药治疗带状疱疹后遗神经痛58例 | 中国麻风皮肤病杂志 | WangDX 2002 | 王德旭 | 青岛市第八人民医院 |
| 血府逐瘀汤加减治疗带状疱疹后遗神经痛临床效果观察 | 皮肤病与性病 | XuGF 2020 | 徐贵芬 | 濮阳市中医医院 |
| 血府逐瘀口服液治疗带状疱疹后遗神经痛30例 | 中国中医药现代远程教育 | LuoJS 2014 | 罗家胜 | 广东省中医院皮肤科 |
| 血府逐瘀汤联合加巴喷丁治疗带状疱疹后遗神经痛34例 | 中国中医药现代远程教育 | ZhangMX 2014 | 张媚霞 | 河北省沧州市沧州和平医院神经内科 |
| 血府逐瘀片辅助治疗带状疱疹后遗神经痛38例 | 中国药业 | MaXJ 2014 | 马晓娟 | 河北省望都皮肤病防治医院 |
| 血府逐瘀汤加减方联合西药治疗带状疱疹后遗神经痛的效果探究 | 当代医药论丛 | HuangBY 2020 | 黄炳炎 | 铜仁市碧江区中医院皮肤科 |
| 血府逐瘀汤加减治疗带状疱疹后神经痛的临床研究 | 健康之友 | DongXL 2019 | 董晓磊 | 长春市中心医院皮肤科 |
| 血府逐瘀汤加减治疗带状疱疹后遗神经痛30例 | 福建中医药 | GuoJH 2017 | 郭建辉 | 龙岩市第二医院 |
| 血府逐瘀汤加减治疗带状疱疹后遗神经痛36例疗效观察 | 中医临床研究 | JiangC 2010 | 江超 | 威海市中医院 |
| 血府逐瘀汤加减治疗带状疱疹后遗神经痛的临床应用意义 | 养生保健指南 | FengC 2021 | 冯程 | 济南市民族医院中医药 |
| 血府逐瘀汤治疗带状疱疹后遗神经痛41例 | 中国中西医结合皮肤性病学杂 | CaiXJ 2007 | 蔡新杰 | 河南省汤阴县人民医院 |
| 延胡止痛方治疗带状疱疹后遗神经痛的疗效观察 | 广州中医药大学学报 | ZhangDH 2020 | 张德华 | 广州中医药大学第四临床医学院 |
| 延胡止痛方治疗带状疱疹后遗神经痛疗效观察 | 深圳中西医结合杂志 | DengFH 2021 | 邓福华 | 长沙市中心医院 |
| 阳和汤加减联合激光治疗带状疱疹后神经痛的临床观察 | 黑龙江中医药大学硕士学位论文 | SunWL 2016 | 孙文磊 | 黑龙江中医药大学 |
| 养血祛瘀法治疗老年带状疱疹后遗神经痛临床观察 | 医学信息 | WuKJ 2016 | 吴科佳 | 常州市中医医院 |
| 一贯煎加减联合甲钴胺与加巴喷丁胶囊治疗带状疱疹后遗神经痛的效果研究 | 中国医学创新 | QinZZ 2022 | 秦珍珍 | 新疆生产建设兵团第十三师红星医院 |
| 益气化瘀止痛汤联合甲钴胺片治疗头部带状疱疹后遗神经痛 64 例疗效观察 | 泰山医学院学报 | SunQQ 2020 | 孙倩倩 | 泰安市中医医院 |
| 益气活血法治疗带状疱疹后遗神经痛27例 | 中国中西医结合皮肤性病学杂志 | GongGF 2014 | 巩国峰 | 山东省广饶县稻庄镇卫生院 |
| 益气活血法治疗老年带状疤疹后遗神经痛30例 | 辽宁中医杂志 | LiXH 2003 | 李秀红 | 朝阳市中心医院中医科 |
| 益气活血化痰汤为主治疗带状疱疹后神经痛疗效观察 | 上海中医药杂志 | ZhangQH 2011 | 张庆华 | 上海中医药大学附属龙华医院麻醉科 |
| 益气活血汤为主治疗带状疱疹后遗神经痛20例 | 河北中医药学报 | ZhangX 2010 | 张欣 | 河北省黄壁庄水库管理局医务室 |
| 益气活血止痛方治疗中老年气滞血型带状疱疹后遗神经痛的临床观察及对血清IL-2水平的影响 | 山东中医药大学学位论文 | LiQR 2010 | 李秋蕊 | 山东中医药大学 |
| 益气活血止痛汤治疗气滞血瘀型带状疱疹后遗神经痛患者的效果 | 中国民康医学 | TianZT 2023 | 田振涛 | 安阳地区医院疼痛科 |
| 益气通络疏肝汤治疗带状疱疹后遗神经痛随机平行对照研究 | 实用中医内科杂志 | GuXQ 2014 | 顾晓群 | 江苏省如皋皋南医院内科 |
| 益气养血扶正法治疗带状疱疹后遗神经痛50例临床观察 | 新中医 | HanXB 2012 | 韩晓冰 | 新疆维吾尔自治区中医院皮肤科 |
| 益气养阴活血法治疗带状疱疹后遗神经痛40例 | 光明中医 | YangPL 2014 | 杨培丽 | 云南昆明市呈贡区人民医院中医科 |
| 鱼腥草减轻老年人带状疱疹后遗神经痛的疗效初探 | 临床皮肤科杂志 | LinZG 2004 | 林子刚 | 第一军医大学南方医院皮肤科， |
| 元胡止痛片联合布洛芬和维生素B1治疗带状疱疹后遗神经痛的疗效观察 | 现代药物与临床 | YuXY 2015 | 俞晓艳 | 上海交通大学医学院附属第三人民医院 |
| 中西医结合治疗带状疱疹后神经痛52例 | 浙江中医杂志 | LiCZ 1999 | 李承珍 | 浙江省湖州市中医院 |
| 中西医结合治疗带状疱疹后神经痛临床观察 | 四川中医 | LuoT 2008 | 罗涛 | 成都中医药大学附属医院疼痛门诊 |
| 中西医结合治疗带状疱疹后遗留神经痛30例疗效观察 | 中国中西医结合杂志 | LiQ 2007 | 李谦 | 昆明医学院第一附属医院皮肤性病科 |
| 中西医结合治疗带状疱疹后遗神52例经痛临床观察 | 黑龙江中医药 | ChaJD 2013 | 查锦东 | 湖北中医药大学临床医学院 |
| 中西医结合治疗带状疱疹后遗神经痛 | 吉林医学 | WanJ 2012 | 万静 | 陕西省延安市人民医院皮肤科 |
| 中西医结合治疗带状疱疹后遗神经痛 | 中国医药指南 | YaoWZ 2015 | 姚文忠 | 陵川县人民医院皮肤科 |
| 中西医结合治疗带状疱疹后遗神经痛24例临床观察 | 中医药导报 | WangHZ 2005 | 汪海珍 | 中建五局职工医院 |
| 中西医结合治疗带状疱疹后遗神经痛30例 | 实用中医药杂志 | CaoGX 2005 | 曹桂熙 | 四川省中医药研究院中医研究所 |
| 中西医结合治疗带状疱疹后遗神经痛40例疗效观察 | 大家健康 | JiaJT 2016 | 贾锦韬 | 陆川县皮肤病防治站皮肤科 |
| 中西医结合治疗带状疱疹后遗神经痛41例 | 现代中西医结合杂志 | YeZM 2008 | 叶正明 | 湖南师范大学附属湘东医院 |
| 中西医结合治疗带状疱疹后遗神经痛43例 | 吉林中医药 | ShenXY 2010 | 沈晓英 | 常州市中医医院 |
| 中西医结合治疗带状疱疹后遗神经痛47例 | 新中医 | MengH 2002 | 孟辉 | 暨南大学医学院中医学系 |
| 中西医结合治疗带状疱疹后遗神经痛48例 | 哈尔滨医药 | ZhuMT 2004 | 朱明田 | 山东省微山县赵庙医院 |
| 中西医结合治疗带状疱疹后遗神经痛42例 | 时珍国医国药 | LiXH 2000 | 李新华 | 山东省莱芜市康复医院 |
| 中西医结合治疗带状疱疹后遗神经痛49例 | 中国民间疗法 | ZhuangZJ 2015 | 庄志江 | 河南中医学院第一附属医院 |
| 中西医结合治疗带状疱疹后遗神经痛50例的临床疗效分析 | 内蒙古中医药 | RuiP 2016 | 芮鹏 | 天津中医药大学 |
| 中西医结合治疗带状疱疹后遗神经痛56例分析 | 中国实用医药 | YuanN 2018 | 袁宁 | 北京市昌平区医院 |
| 中西医结合治疗带状疱疹后遗神经痛60例的疗效观察 | 内蒙古中医药 | GuoYL 2016 | 郭雅莉 | 天津市海河医院 |
| 中西医结合治疗带状疱疹后遗神经痛60例疗效观察 | 山东中西医结合皮肤性病学术会议 | WangKJ 2011 | 王奎军 | 平度市皮肤病防治站 |
| 中西医结合治疗带状疱疹后遗神经痛60例疗效观察 | 新中医 | JiangLY 2014 | 江丽莹 | 广州市东升医院 |
| 中西医结合治疗带状疱疹后遗神经痛63例疗效观察 | 新中医 | LanWM 2015 | 兰卫明 | 景宁族自治县人民医院 |
| 中西医结合治疗带状疱疹后遗神经痛78例 | 中国疗养医学2009 | LiuCH 2009 | 刘春华 | 兰州军区临潼疗养院 |
| 中西医结合治疗带状疱疹后遗神经痛105例疗效观察 | 河北中医 | YuanSZ 2011 | 苑淑尊 | 冀中能源邢矿集团有限责任公司总医院皮肤科 |
| 中西医结合治疗带状疱疹后遗神经痛128例 | 皮肤病与性病 | MaLM 2005 | 马黎民 | 河南省确山卫校皮肤科 |
| 中西医结合治疗带状疱疹后遗神经痛的疗效分析 | 医学理论与实践 | WangBL 2015 | 汪本龙 | 安徽省歙县第二人民医院内科 |
| 中西医结合治疗带状疱疹后遗神经痛的临床观察 | 中国保健营养 | ChangJY 2020 | 常加银 | 云南省大理州南涧县宝华镇中心卫生院中医科 |
| 中西医结合治疗带状疱疹后遗神经痛的临床观察 | 中国保健营养 | LuoF 2018 | 罗锋 | 宁夏固原市原州区社保局 |
| 中西医结合治疗带状疱疹后遗神经痛的临床疗效 | 世界临床医学 | TianHL 2017 | 田汇林 | 湖北省蕲春县人民医院神经内科 |
| 中西医结合治疗带状疱疹后遗神经痛的临床疗效分析 | 国际医药卫生导报 | ChenSW2006 | 陈素文 | 佛山市南海区妇幼保健院 |
| 中西医结合治疗带状疱疹后遗神经痛疗效分析 | [养生保健指南](http://sns.wanfangdata.com.cn-s.dres.hactcm.edu.cn/perio/ysbjzn-x) | LiLQ 2018 | 李丽琼 | 云南省昆明市五华区人民医院 |
| 中西医结合治疗带状疱疹后遗神经痛的研究 | 中医临床杂志 | ZhangJ 2013 | 张娟 | 山东医学高等专科学校 |
| 元胡止痛片联合普瑞巴林治疗带状疱疹后遗神经痛的临床研究 | 现代药物与临床 | ZhangP 2021 | 张沛 | 河南科技大学第一附属医院 |
| 元通合剂、阿昔洛韦联合氦氖激光治疗带状疱疹的临床疗效观察 | 湖北中医学院硕士学位论文 | YeYY 2007 | 叶缘苑 | 湖北中医学院 |
| 珍宝丸结合多虑平片治疗带状疱疹后遗神经痛疗效观察 | 中华中医药杂志 | YeJJ 2015 | 叶静静 | 宁波市中医院皮肤科 |
| 正清风痛宁缓释片联合加巴喷丁胶囊治疗亚急性期 带状疱疹后神经痛的临床观察 | 中国疼痛医学杂志 | WangM 2021 | 王萌 | 南昌大学第一附属医院疼痛科 |
| 正清风痛宁缓释片联合普瑞巴林治疗带状疱疹后遗神经痛的临床研究 | 现代药物与临床 | WangJ 2022 | 王静 | 河南科技大学第一附属医院景华院区 |
| 止痛颗粒治疗带状疱疹后遗神经痛112例临床观察 | 河北中医 | LiXP 2005 | 李秀萍 | 河北省石家庄市中医院 |
| 止痛如神汤加减治疗带状疱疹后遗神经痛的临床效果 | 中西医结合研究 | ChenDJ 2016 | 陈德监 | 江苏省兴化市中医院皮肤科 |
| 止痛饮治疗带状疱疹后遗神经痛42例 | 陕西中医 | TanCM 2004 | 谭春明 | 广东省佛山市顺德中西医结合医院 |
| 中西药合用治疗带状疱疹后神经痛临床观察 | 实用中医药杂志 | WangLF 2019 | 王来福 | 河南省驻马店市中心医院疼痛科 |
| 中西药合用治疗带状疱疹后遗神经痛 对疼痛及炎性因子的影响 | 实用中医药杂志 | WangAJ 2020 | 王爱军 | 江苏省常州市新北区三井人民医院皮肤科 |
| 中西药合用治疗带状疱疹后遗神经痛临床观察 | 实用中医药杂志 | DouCH 2018 | 窦晨辉 | 河南省郑州人民医院中医科 |
| 中西药结合治疗带状疱疹后遗神经痛对比观察 | 河北医学 | YinDY 2003 | 印丹悦 | 江苏省如皋市中医院 |
| 中西药联合红光治疗带状疱疹后遗神经痛120例的疗效观察 | 世界最新医学信息文摘 | CaiSL 2019 | 蔡思龙 | 武汉科技大学医院皮肤科 |
| 中西药物结合治疗带状疱疹后遗神经痛128例 | 社区中医药 | GuoWH 2008 | 郭文华 | 吉林省吉林市高新区郭文华皮肤科诊所 |
| 中西医结合对带状疱疹后神经痛疗效、睡眠质量及情绪障碍分析 | 中华中医药学刊 | ShenY 2016 | 沈涌 | 杭州市中医院神经内科 |
| 治疗带状疱疹后遗神经痛60例报告 | 江西中医药 | BaoYS 2000 | 鲍云生 | 浙江省台州市中医院 |
| 中西结合治疗带状疱疹后遗神经痛的疗效及对免疫功能的调节 | 中医中药 | YangC 2013 | 杨超 | 湖南省娄底市中心医院皮肤性病科 |
| 中西药结合治疗带状疱疹后遗神经痛疗效观察 | 中国中西医结合皮肤性病学杂志 | SunCW 2012 | 孙春秋 | 杭州市第三人民医院 |
| 中西医结合疗法治疗带状疱疹后遗神经痛 | 内蒙古医学杂志 | HeJZ 2014 | 何金柱 | 内蒙古自治区人民医院 |
| 中西医结合治疗带状疱疹后遗神经痛疗效观察 | 新中医 | LuoSP 2010 | 罗水平 | 佛山市南海区九江医院皮肤科 |
| 中西医结合治疗带状疱疹后遗神经痛疗效观察 | 实用中西医结合临床 | HouBM 2010 | 侯保民 | 河南省周口市第五人民医院 |
| 中西医结合治疗带状疱疹后遗神经痛疗效观察 | 时珍国医国药 | YanH 2022 | 严欢 | 湖北省直属机关医院／湖北省康复医院 |
| 中西医结合治疗带状疱疹后遗神经痛疗效观察 | 健康必读 | GuanQL 2019 | 管庆玲 | 陕西省汉中市汉台区七里中心卫生院 |
| 中西医结合治疗带状疱疹后遗神经痛疗效观察 | 健康医学 | WanHL 2009 | 万卉蕾 | 郑州人民医院 |
| 中西医结合治疗带状疱疹后遗神经痛疗效观察 | 现代中西医结合杂志 | ChenYM 2016 | 陈玉梅 | 湖北省恩施市中心医院 |
| 中西医结合治疗带状疱疹后遗神经痛疗效观察 | 航空航天医学杂志 | WangJC 2018 | 王金蝉 | 贵航302医院 |
| 中西医结合治疗带状疱疹后遗神经痛疗效观察 | 临床军医杂志 | ZhangY 2017 | 张莹 | 沈阳军区总医院 |
| 中西医结合治疗带状疱疹后遗神经痛疗效评价 | 中国保健营养 | JinZ 2016 | 金枝 | 云南省丽江市永胜县中医院内一科 |
| 中西医结合治疗带状疱疹后遗神经痛临床分析 | 中国民康医学 | TongL 2008 | 童璐 | 沈阳市第七人民医院皮肤科 |
| 中西医结合治疗带状疱疹后遗神经痛临床观察 | 中国中医药现代远程教育 | DengAH 2021 | 邓爱华 | 崇仁县马鞍镇中心卫生院中医科 |
| 中西医结合治疗带状疱疹后遗神经痛临床观察 | 湖北中医杂志 | YeZW 2009 | 叶志义 | 湖北省崇阳县中医院 |
| 中西医结合治疗带状疱疹遗留神经痛40例临床观察 | 中国学校卫生 | YuanHY 2008 | 袁惠英 | 南京农业大学医院 |
| 中西医结合治疗带状疱疹后遗神经痛疗效观察 | 职业与健康 | ZHangH 2002 | 张辉 | 山东省肥城市人民医院 |
| 中西医结合治疗老年带状疱疹后遗神经痛40例 | 福建中医药 | RuanAX 2000 | 阮爱星 | 福建省皮肤病性病防治院 |
| 肿痛安胶囊加音频电疗治疗带状疱疹后遗神经痛 | 中国误诊学杂志 | XiaYH 2008 | 夏永华 | 新乡医学院第一附属医院皮肤性病科 |
| 自拟除湿胃苓汤加减治疗带状疱疹神经痛30例疗效观察 | 疑难病杂志 | ZhangGP 2012 | 张桂萍 | 北京中医药大学附属护国寺中医医院 |
| 自拟虎杖雄黄散合复元活血汤治疗带状疱疹后遗神经痛疗效观察 | 九江学院学报(自然科学版) | ZhouCL 2015 | 周春来 | 共青城市人民医院中医科 |
| 自拟化瘀止痛汤联合普瑞巴林胶囊治疗带状疱疹后神经痛气滞血瘀证的疗效观察 | 中国中医药科技 | QuYX 2022 | 曲亚鑫 | 黑龙江省中医药科学院 |
| 自拟活血柔肝汤治疗带状疱疹后遗神经痛疗效观察 | 内蒙古中医药 | ZhongJ 2001 | 钟江 | 广西中医学院第一附属医院 |
| 自拟活血散瘀汤治疗带状疱疹后遗神经痛疗效观察 | 光明中医 | ZhangL 2011 | 张玲 | 山西省中医院 |
| 自拟活血通络止痛汤加减治疗带状疱疹后遗肋间神经痛的临床观察 | 临床和实验医学杂志 | ZhangBH 2015 | 张保恒 | 甘肃省武威市人民医院皮肤科 |
| 自拟解郁清毒散治疗带状疱疹后遗神经痛伴焦虑抑郁疗效观察 | 临床合理用药 | LongXC 2019 | 龙雄初 | 北京市朝阳区潘家园第二社区卫生服务中心内科 |
| 自拟理气活血方治疗75例带状疱疹后遗神经痛疗效观察 | 中医临床研究 | HuYG 2012 | 胡艳阁 | 河南省传染病医院 |
| 自拟疱疹止痛汤加减治疗老年带状疱疹后神经痛疗效观察 | 中国误诊学杂志 | KongYS 2011 | 孔玉沙 | 河南省周口市中心医院皮肤性病科 |
| 自拟四虫益气养血汤联合普瑞巴林治疗带状疱疹后遗神经痛的临床疗效 | 中国现代医生 | XunL 2021 | 孙岚 | 湖北省荆州市第三人民医院皮肤科 |
| 自拟通络益气方辅助治疗80例顽固性带状疱疹后遗神经痛对血清炎性因子、免疫球蛋白含量的影响分析 | 四川中医 | WuWM 2018 | 吴为民 | 廊坊市第四人民医院 |
| 自拟通蝎汤治疗带状疱疹后遗神经痛疗效观察 | 广西中医药 | LuoW 2007 | 罗威 | 梧州市中西医结合医院 |
| 自拟益气活血汤治疗带状疱疹后遗神经痛临床分析 | 中国中西医结合皮肤性病学杂志 | ZhouM 2014 | 周蜜 | 浙江省绍兴市第六人民医院 |
| 自拟益气通络方治疗中老年带状疱疹后遗神经痛的临床疗效评价 | 自我保健 | GuJH 2023 | 顾景辉 | 北京市第一中西医结合医院治未病科 |
| 自拟止痛汤治疗带状疱疹后遗神经痛48例临床观察 | 浙江中医杂志 | ZhangMF 2015 | 张明峰 | 浙江省湖州市中医院 |
| 自拟中药疱疹止痛灵治疗中老年带状疱疹后遗神经痛的临床疗效 | 中国老年学杂志 | ZhangEL 2014 | 张二力 | 吉林大学白求恩第一医院 |
| 自拟中药疱疹止痛灵治疗中老年带状疱疹后遗神经痛的 临床疗效 | 中国生化药物杂志 | LeiJG 2017 | 雷进功 | 绥德县中医医院中医皮肤科 |
| 中西医结合治疗老年带状疱疹后遗神经痛240 例疗效观察 | 河北中医 | ZhaoCJ 2009 | 赵存杰 | 河北省邯郸市第一医院中西医结合内科 |
| 中西医结合治疗老年带状疱疹神经痛 | 国际中医中药杂志 | GeSH 2008 | 葛苏华 | 北京世纪坛医院 |
| 中西医结合治疗老年人带状疱疹后遗神经痛临床疗效观察 | 中国老年保健医学 | XiYT 2010 | 奚燕萍 | 昆明市中医院 |
| 中西医治疗带状疱疹后遗神经痛的疗效分析 | 中医临床研究 | GaoWY 2014 | 高文有 | 北京汉典中医研究院 |
| 中西医治疗带状疱疹后遗神经痛对照观察 | 实用心脑肺血管病杂志 | LiJJ 2012 | 李静军 | 郸县郸筒镇社区卫生服务中心 |
| 中药辨证施治联合加巴喷丁治疗带状疱疹后遗神经痛临床观察 | 新中医 | ZhangSY 2018 | 张玉松 | 广州中医药大学附属新会中医院 |
| 中药辅助治疗带状疱疹后遗神经痛 | 中草药 | YangLJ 2010 | 杨丽姣 | 杭州市第三人民医院 |
| 中药联合伐昔洛韦片治疗带状疱疹后遗神经痛疗效观察 | [中国处方药](https://navi.cnki.net/knavi/journals/ZGCF/detail?uniplatform=NZKPT) | WangJF 2018 | 王金凤 | 汪清县中医院皮肤科 |
| 中药祛痛汤结合多虑平治疗带状疱疹后遗神经痛疗效观察 | 检验医学与临床 | HuangJQ 2014 | 黄俊青 | 广东省深圳市宝安区西乡人民医院皮肤科 |
| 中药治疗带状疱疹后遗神经痛80例 | 实用中医药杂志 | ZhuQJ 2002 | 朱其杰 | 广州中医药大学第一附属医院 |
| 中药治疗带状疱疹后遗神经痛临床观察 | 广东省第五届皮肤性病防治学术会议论文汇编 | DongH 2006 | 董海 | 解放军第四五八医院皮肤科 |
| 中药治疗下肢带状疱疹神经痛临床研究 | 中医学报 | WuSD 2014 | 武水斗 | 漯河医学高等专科学校第二附属医院 |
| 中医药治疗老年带状疱疹疼痛68例疗效观察 | 湖南中医药导报 | JiangCZ 2002 | 蒋成章 | 湖南省人民医院 |
| 中医治疗带状疱疹后遗神经痛40例临床观察 | Chinese Journal of Ethnomedicine and Ethnopharmacy | WeiBX 2015 | 魏宝兴 | 广西骨伤医院皮肤科 |
| 中医治疗带状疱疹后遗神经痛70例疗效观察 | 云南中医中药杂志 | WangH 2009 | 王恒 | 上海交通大学附属仁济医院崇明分院 |
| 中医治疗带状疱疹后遗神经痛疗效观察 | 现代中西医结合杂志 | YangLL 2011 | 杨丽莉 | 河南省偃师市中医院 |
| 龙胆泻肝汤联合普瑞巴林治疗带状疱疹后遗神经痛的效果 | 临床医学 | WangQH 2023 | 王庆华 | 山东省济南市钢城区人民医院皮肤科 |
| 逍遥散联合情志疗法治疗带状疱疹后遗神经痛的临床疗效观察 | 湖北中医药大学硕士学位论文 | JuF 2019 | 琚凡 | 湖北中医药大学 |
| 益气活血方配合氯胺酮椎管内阻滞治疗57例顽固性带状疱疹后遗神经痛 | 中国实验方剂学杂志 | FengHJ 2013 | 冯海军 | 苏州九龙医院 |
| 元胡止痛滴丸联合加巴喷丁胶囊治疗带状疱疹后遗神经痛对患者症状的改善评价 | 现代诊断与治疗 | SongYP 2023 | 宋永平 | 上高县人民医院 |
| 中老年带状疱疹后遗神经痛的疗效观察 | 北京中医药 | ZangX 2008 | 臧鑫 | 广东省人民医院 |
| 中西医结合治疗带状疱疹后遗神经痛疗效观察 | 中华中医药杂志 | BaoLX 2009 | 鲍丽霞 | 浙江省武义县中医院 |
| 自拟解郁清毒散治疗带状疱疹后遗神经痛42例临床疗效观察 | 世界中西医结合杂志 | LongXC 2013 | 龙雄初 | 中国人民解放军第二五一医院 |
| 复元活血汤治疗带状疱疹后遗神经痛的效果分析 | 常州实用医学 | ZhouXY 2019 | 周晓芸 | 苏州市中医医院皮肤科 |
| 活血化瘀法治疗老年带状疱疹后遗神经痛的临床效果 | 健康必读 | ZhouTF 2019 | 周庭芳 | 贵州省关岭县自治县中医院 |
| 疱疹止痛灵治疗带状疱疹后遗神经痛的临床与实验研究 | 白求恩医科大学学报 | LiYT 1999 | 李有田 | 第一临床学院中医科 |
| 芍药甘草汤加味治疗带状疱疹后遗神经痛疗效观察 | 新中医 | XiaoWM 2013 | 肖卫棉 | 肇庆市中医院皮肤科 |
| 通络益气方配合氯胺酮椎管内阻滞治疗顽固性带状疱疹后遗神经痛效果观察 | 白求恩医学杂志 | WuWM 2019 | 吴为民 | 河北省廊坊市第四人民医院麻醉科 |
| 五味消毒饮联合龙胆泻肝汤对带状疱疹患者后遗神经痛及IL-6、IL-10的影响分析 | 当代医学 | FengH 2019 | 冯海 | 北京市朝阳区六里屯社区卫生服务中心中医科 |
| 止痛汤联合威伐光治疗带状疱疹后神经痛的临床观察 | 黑龙江中医药大学硕士学位论文 | SunYR 2019 | 孙艺榕 | 黑龙江中医药大学 |
| 中西医结合治疗带状疱疹后遗神经痛临床观察 | 医学理论与实践 | ZhaoJH 2013 | 赵继华 | 天津市蓟县中医医院皮肤科 |
| 补气活血法治疗带状疱疹后神经痛疗效观察 | 实用中医药杂志 | SunYW 2023 | 孙有为 | 北京市丰台区西罗园社区卫生服务中心中医康复科 |
| 身痛逐瘀汤治疗气滞血瘀型带状疱疹后遗神经痛临床观察 | 光明中医 | NiGY 2023 | 倪国勇 | 北京市东城金针研究学会海运仓中医门诊部中医科 |
| 复元活血汤治疗带状疱疹后遗神经痛53例 | 陕西中医 | ZhangL 2013 | 张良 | 四川省乐山市第四人民医院皮肤科 |
| 神经阻滞联合复元活血汤治疗带状疱疹后神经痛28例 | 中国中医药信息杂志 | ZhangYZ 2009 | 张友芝 | 浏阳市集里医院疼痛专科 |
| 血府逐瘀汤合金铃子散治疗带状疱疹后遗神经痛174例 | 山东中医杂志 | LiZM 1998 | 李政敏 | 平度市人民医院 |
| 青黛五虫丸治疗带状疱疹后遗神经痛62例 | 陕西中医 | LiXX 1999 | 李学兴 | 浙江省兰溪市中医院 |
| 中药联合火针治疗气虚血瘀型带状疱疹后遗神经痛的临床观察 | 黑龙江中医药大学硕士学位论文 | ZhaoWZ 2023 | 赵文振 | 黑龙江中医药大学 |
| 止痛汤联合威伐光治疗带状疱疹性神经痛的疗效观察 | 中医与中药 | HuYS 2023 | 胡艳双 | 黑龙江省牡丹江市第一人民医院皮肤性病科 |
| 川芎茶调散加减联合普瑞巴林治疗头面部带状疱疹后遗神经痛的临床疗效观察 | 湖北中医药大学硕士学位论文 | ZhanXY 2023 | 湛晓娅 | 湖北中医药大学 |
| 自拟益气止痛方治疗气虚血瘀型带状疱疹后神经痛的临床疗效观察 | 江西中医药大学硕士学位论文 | WenJP 2023 | 文佳鹏 | 江西中医药大学 |
| 草乌甲素片联合利多卡因凝胶贴膏治疗带状疱疹后神经痛的临床研究 | 现代药物与临床 | ZhaoL 2024 | 赵蕾 | 河南科技大学第一附属医院 疼痛科 |
| 柴胡疏肝散联合阶梯针刺法对气滞血瘀型带状疱疹后遗神经痛患者的影响 | 四川中医 | GuoHM 2024 | 郭慧敏 | 延安市中医医院皮肤科 |
| 柴胡疏肝散联合桃红四物汤加减治疗带状疱疹后遗神经痛临床研究 | 新中医 | XieZQ 2024 | 解增强 | 长兴县人民医院皮肤科 |
| 大柴胡汤联合针灸治疗带状疱疹后神经痛的临床疗效 | 深圳中西医结合杂志 | YuF 2024 | 于飞 | 佳木斯中心医院 |
| 观察柴胡疏肝散合桃红四物汤加减治疗带状疱疹后遗神经痛的临床疗效 | 中文科技期刊数据库（文摘版）医药卫生 | HuangZH 2023 | 黄自华 | 柳州市柳北区沙塘镇中心卫生院 |
| 理气治血方联合西医治疗气滞血瘀型带状疱疹后遗神经痛疗效研究 | 中国科技期刊数据库医药 | TanXH 2024 | 谭晓慧 | 中江县人民医院 |
| 身痛逐瘀汤合四逆散治疗带状疱疹后遗神经痛的临床疗效观察 | 中国处方药 | ZhuY 224 | 朱英 | 昆山市中西医结合医院疼痛科 |
| 双莲蛇草汤对带状疱疹后遗神经痛患者疼痛状况及心理状况的影响 | 中华中医药学刊 | ZhuGJ 2024 | 朱国佳 | 河北中医药大学研究生院 |
| 小柴胡汤合瓜蒌红花散加减联合常规西药治疗带状疱疹后遗神经痛的效果观察 | 社区中医药 | WangF 2023 | 王菲 | 北京市东城区天坛社区卫生服务中心 |
| 小柴胡汤合桔梗甘草汤治疗带状疱疹神经痛的疗效观察 | 医学理论与实践 | ZhouHX 2024 | 周辉霞 | 广东省广州市固生堂岭南中医馆东山门诊部 |
| 血府逐瘀胶囊联合普瑞巴林胶囊对带状疱疹后遗神经痛患者疼痛及血清炎症因子水平的影响 | 反射疗法与康复医学 | JingXY 2024 | 井祥云 | 五莲县人民医院皮肤科 |
| 血府逐瘀汤加减联合普瑞巴林治疗带状疱疹后神经痛（气滞血瘀型）的临床疗效观察 | 湖北中医药大学硕士学位论文 | ChenC 2024 | 陈聪 | 湖北中医药大学 |
| 长桑君脉法脉息术指导下大柴胡汤治疗带状疱疹后遗神经痛的效果评价 | 中文科技期刊数据库（全文版）医药卫生 | YaoBB 2023 | 姚彬彬 | 临清市中医医院 |
| 止痛如神汤在腰骶部带状疱疹后神经痛（脾虚湿蕴）患者中的应用及对疼痛程度的影响 | 中外医学研究 | HongY 2024 | 洪勇 | 彭州市人民医院 |
| 中西医结合治疗带状疱疹后遗神经痛临床观察 | 山西中医 | ZhouP 2024 | 周鹏 | 湖北中医药大学 |
| 中药辨证施治联合超声引导下椎旁神经阻滞治疗带状疱疹后神经痛的疗效 | 辽宁中医药大学学报 | MiYP 2023 | 米永鹏 | 石家庄市中医院 |
| 中药补阳还五汤结合脉冲射频治疗带状疱疹后神经痛的效果 | 中文科技期刊数据库（全文版）医药卫生 | LiB 2023 | 李宾 | 北京市健宫医院疼痛科 |
| 氨酚曲马多联合中药治疗带状疱疹后遗神经痛的疗效观察 | 中外健康文摘 | ShiQ 2011 | 石全 | 湖北省中医院皮肤科 |
| 补阳还五汤结合脉冲射频治疗带状疱疹后神经痛的临床观察 | 上海中医药大学硕士学位论文 | GuAN 2017 | 顾艾娜 | 上海中医药大学 |
| 柴胡疏肝散合桃红四物汤治疗带状疱疹后遗神经痛疗效观察 | 中外健康文摘 | JinZ 2008 | 金忠 | 成都中医药大学 |
| 香丹止痛方联合西药治疗肝郁气滞血瘀PHN患者的疗效观察及对Th17/Treg细胞平衡的影响 | 中国中西医结合杂志 | ChenL 2019 | 陈琳 | 福建中医药大学附属人民医院疼痛科 |
| 血府逐瘀汤加四妙勇安汤化裁方治疗带状疱疹后遗神经痛的临床研究 | 中华现代中西医杂志 | ZhuPC 2004 | 朱培成 | 广东省广州市红十字会医院皮肤科 |
| 中药联合甲钴胺治疗顽固性带状疱疹后遗性神经痛 | 中国临床康复 | TianZW 2005 | 田中伟 | 新乡医学院基础医学院免疫学教研室 |
