# Supplementary material for: Randomized controlled trials of major oral traditional Chinese medicine preparations for postherpetic neuralgia: an evidence map
Source: Front Pharmacol. 2026 Jun 8;17:1815376. doi: 10.3389/fphar.2026.1815376 (PMC13311778; doi:10.3389/fphar.2026.1815376)
Supplement: Supplementary file 3 [file DataSheet2.docx]

**The search strategy of PubMed database**

**#1** Search: Neuralgia, Postherpetic[MeSH Terms]

**#2** Search: (((((((postherpetic neuralgia[Title/Abstract]) OR (Postherpetic neuralgia[Title/Abstract])) OR (PHN[Title/Abstract])) OR (post herpetic neuralgia[Title/Abstract])) OR (post-herpetic neuralgia[Title/Abstract])) OR (postherpetic pain[Title/Abstract])) OR (post herpetic pain[Title/Abstract])) OR (post-herpetic pain[Title/Abstract])

**#3** Search: (#1) OR (#2)

**#4** Search: (Medicine, Chinese Traditional[MeSH Terms]) OR (Drugs, Chinese Herbal[MeSH Terms])

**#5** Search: ((((((Chinese Medicine[Title/Abstract]) OR (Chinese Drug[Title/Abstract])) OR (Traditional Herb[Title/Abstract])) OR (Traditional Drug[Title/Abstract])) OR (Traditional Medicine[Title/Abstract])) OR (Chinese patent drug[Title/Abstract])) OR (injection[Title/Abstract])

**#6** Search: (((((huoxue[Title/Abstract]) OR (zhitong[Title/Abstract])) OR (tongluo[Title/Abstract])) OR (zhuyu[Title/Abstract])) OR (fuzheng[Title/Abstract])) OR (liver[Title/Abstract])

**#7** Search: (#4) OR (#5) OR (#6)

**#8** Search: (#3) AND (#7)

**The search strategy of the Cochrane Library database**

**#1** MeSH descriptor: [Neuralgia, Postherpetic] explode all trees

**#2** (postherpetic neuralgia):ti,ab,kw OR (Postherpetic neuralgia):ti,ab,kw OR (PHN):ti,ab,kw OR (post herpetic neuralgia):ti,ab,kw OR (post-herpetic neuralgia):ti,ab,kw OR ('postherpetic pain):ti,ab,kw OR (post herpetic pain):ti,ab,kw OR (post-herpetic pain):ti,ab,kw

**#3** #1 OR #2

**#4** MeSH descriptor: [Medicine, Chinese Traditional] explode all trees

**#5** MeSH descriptor: [Drugs, Chinese Herbal] explode all trees

**#6** (Chinese Medicine):ti,ab,kw OR (Chinese Drug):ti,ab,kw OR (Traditional Herb):ti,ab,kw OR (Traditional Drug):ti,ab,kw OR (Traditional Medicine):ti,ab,kw OR (Chinese patent drug):ti,ab,kw OR (injection):ti,ab,kw

**#7** (huoxue):ti,ab,kw OR (zhitong):ti,ab,kw OR (tongluo):ti,ab,kw OR (zhuyu):ti,ab,kw OR (fuzheng):ti,ab,kw OR (liver):ti,ab,kw

**#8** #4 OR #5 OR #6 OR #7

**#9** #3 AND #8

**The search strategy of Embase database**

**#1** 'postherpetic neuralgia'/exp

**#2** 'Postherpetic neuralgia':ti,ab OR 'PHN':ti,ab OR 'postherpetic neuralgia':ti,ab OR 'post herpetic neuralgia':ti,ab OR 'post-herpetic neuralgia':ti,ab OR 'postherpetic pain':ti,ab OR 'post herpetic pain':ti,ab OR 'post-herpetic pain':ti,ab

**#3** #1 OR #2

**#4** ‘Chinese medicine’/exp OR ‘Chinese drug’/exp

**#5** ‘Chinese Medicine’**:ti,ab OR ‘**Chinese Drug’**:ti,ab OR ‘**Traditional Herb’**:ti,ab OR ‘**Traditional Drug’**:ti,ab OR ‘Chinese Medicine’:ti,ab OR ‘**Moxibustion’**:ti,ab OR ‘Chinese Drug’:ti,ab OR ‘Chinese Herb’:ti,ab OR ‘Traditional Medicine’:ti,ab OR ‘Chinese patent drug’:ti,ab OR ‘injection’:ti,ab**

**#6** ‘huoxue’:ti,ab OR ‘zhitong’:ti,ab OR ‘tongluo’:ti,ab OR ‘zhuyu’:ti,ab OR ‘fuzheng’:ti,ab OR ‘liver’:ti,ab

**#7** #4 OR #5 OR #6

**#8** #3 AND #7

**The search strategy of China National Knowledge Infrastructure (CNKI) database**

TKA=带状疱疹后遗神经痛+带状疱疹后神经痛+postherpetic neuralgia+缠腰火丹+蛇串疮 AND TKA=中医+中医药+中药+中西医结合+中西医联合+中药制剂+中药内服+中药汤剂+饮片+中药疗法+联合中药+口服中药+传统中药+自拟+中成药+中草药+复方+煎+汤+丸+膏+散+露+丹+方+剂+药+活血+止痛+通络+逐瘀+扶正+肝

**The search strategy of Wanfang Data**

主题: (("带状疱疹后遗神经痛"or"带状疱疹后神经痛"or"postherpetic neuralgia"or"缠腰火丹"or"蛇串疮") AND ("中医"or"中医药"or"中药"or"中西医结合"or"中西医联合"or"中药制剂"or"中药内服"or"中药汤剂"or"饮片"or"中药疗法"or"联合中药"or"口服中药"or"传统中药"or"自拟"or"中成药"or"中草药"or"复方"or"煎"or"汤"or"丸"or"膏"or"散"or"露"or"丹"or"方剂"or"活血"or"止痛"or"通络"or"逐瘀"or"肝"))

**The search strategy of VIP Database**

M=((带状疱疹后遗神经痛 or 带状疱疹后神经痛 or postherpetic neuralgia or 缠腰火丹 or 蛇串疮) AND (中医 or 中医药 or 中药 or 中西医结合 or 中西医联合 or 中药制剂 or 中药内服 or 中药汤剂 or 饮片 or 中药疗法 or 联合中药 or 口服中药 or 传统中药 or 自拟 or 中成药 or 中草药 or 复方 or 煎 or 汤 or 丸 or 膏 or 散 or 露 or 丹 or 方 or 剂 or 药 or 活血 or 止痛 or 通络 or 逐瘀 or 扶正 or 肝))

**The search strategy of China Biomedical Literature Database (CBM)**

("带状疱疹后遗神经痛" [核心字段:智能] OR "带状疱疹后神经痛" [核心字段:智能] OR "postherpetic neuralgia" [核心字段:智能] OR "缠腰火丹" [核心字段:智能] OR "蛇串疮" [核心字段:智能]) AND ("中医" [核心字段:智能] OR "中医药" [核心字段:智能] OR "中药" [核心字段:智能] OR "中西医结合" [核心字段:智能] OR "中西医联合" [核心字段:智能] OR "中药制剂" [核心字段:智能] OR "中药内服" [核心字段:智能] OR "中药汤剂" [核心字段:智能] OR "饮片" [核心字段:智能] OR "中药疗法" [核心字段:智能] OR "联合中药" [核心字段:智能] OR "口服中药" [核心字段:智能] OR "传统中药" [核心字段:智能] OR "自拟" [核心字段:智能] OR "中成药" [核心字段:智能] OR "中草药" [核心字段:智能] OR "复方" [核心字段:智能] OR "煎" [核心字段:智能] OR "汤" [核心字段:智能] OR "丸" [核心字段:智能] OR "膏" [核心字段:智能] OR "散" [核心字段:智能] OR "露" [核心字段:智能] OR "丹" [核心字段:智能] OR "方" [核心字段:智能] OR "剂" [核心字段:智能] OR "药" [核心字段:智能] OR "活血" [核心字段:智能] OR "止痛" [核心字段:智能] OR "通络" [核心字段:智能] OR "逐瘀" [核心字段:智能] OR "扶正" [核心字段:智能] OR "肝" [核心字段:智能])
